# *Paracoccidioides* Genomes Reflect High Levels of Species Divergence and Little Interspecific Gene Flow

**DOI:** 10.1128/mBio.01999-20

**Published:** 2020-12-22

**Authors:** Heidi Mavengere, Kathleen Mattox, Marcus M. Teixeira, Victoria E. Sepúlveda, Oscar M. Gomez, Orville Hernandez, Juan McEwen, Daniel R. Matute

**Affiliations:** a Biology Department, University of North Carolina, Chapel Hill, North Carolina, USA; b Núcleo de Medicina Tropical, Faculdade de Medicina, University of Brasília, Brasília, Brazil; c Department of Microbiology and Immunology, University of North Carolina, Chapel Hill, North Carolina, USA; d Cellular and Molecular Biology Unit, Corporación para Investigaciones Biológicas, Medellín, Colombia; e MICROBA Research Group, School of Microbiology, Universidad de Antioquia, Medellín, Colombia; f School of Medicine, Universidad de Antioquia, Medellín, Colombia; Vallabhbhai Patel Chest Institute

**Keywords:** speciation, gene exchange, hidden Markov model (HMM), introgression

## Abstract

The fungus Paracoccidioides is a prevalent human pathogen endemic to South America. The genus is composed of five species. In this report, we use 37 whole-genome sequences to study the allocation of genetic variation in *Paracoccidioides*. We tested three genome-wide predictions of advanced speciation, namely, that all species should be reciprocally monophyletic, that species pairs should be highly differentiated along the whole genome, and that there should be low rates of interspecific gene exchange. We find support for these three hypotheses. Species pairs with older divergences show no evidence of gene exchange, while more recently diverged species pairs show evidence of modest rates of introgression. Our results indicate that as divergence progresses, species boundaries become less porous among *Paracoccidioides* species. Our results suggest that species in *Paracoccidioides* are at different stages along the divergence continuum.

## INTRODUCTION

*Paracoccidioides*, a genus of temperature-dimorphic fungi, causes paracoccidioidomycosis (PCM), which is a systemic endemic mycosis that occurs across in most countries of Latin America from mideastern Mexico to Argentina (1, 2). Multiple genetic surveys have revealed extensive genetic variability within *Paracoccidioides* (3–7). This variation, coupled with the extensive geographic range of the fungus—and that of the disease it causes—led to the hypothesis of population structure and cryptic speciation within the group. Initial studies reveal the existence of at least three species (8), but more recent analyses have suggested the existence of five different species of *Paracoccidioides* (9–11). Clearly, the use of genetic markers holds the potential to reveal key aspects of the evolutionary biology of the pathogen. Yet, the genetic characterization of most isolates has been modest.

Species from the genus *Paracoccidioides* show a range of divergence that make the group promising to understand how diversification occurs in pathogenic fungi. One of the species of *Paracoccidioides*, Paracoccidioides lutzii, seems to have diverged from the rest of the species at least 30 million years ago (12). The species show extensive differences in terms of morphology and physiology. Five more species, all within the *brasiliensis* complex, form a monophyletic group. Paracoccidioides restrepiensis and Paracoccidioides venezuelensis are the most closely related dyad with recent divergence (less than 0.2 million years ago [MYA]) (10). Paracoccidioides brasiliensis *sensu stricto* is sister to the *P. restrepiensis/P. venezuelensis* dyad, while Paracoccidioides americana is sister to the ingroup. Paracoccidioides brasiliensis *sensu stricto* has also been proposed to be formed by two cryptic species, S1a and S1b (13), but no formal test of this divergence has been performed. A combination of yeast and conidial morphology differentiates between all *Paracoccidioides* species pairs (10, 11, 14).

Whole-genome sequences can be used to identify species boundaries in fungi. Three tests jointly indicate the existence of species boundaries (15). First, genome variation must reflect genetic differentiation in cases where speciation has taken place. In cases of advanced divergence, genomes of putative species should show reciprocal monophyly; this can be measured as the proportion of loci that show a phylogenetic history concordant with the hypothesized species history. Second, genetic variation should be partitioned across putative species; the extent of genetic differentiation between individuals from different putative species should be larger than the differentiation between individuals within each of the putative species. Finally, the genomes of putative species should show low or moderate levels of gene exchange. So far, all studies on species boundaries in the genus *Paracoccidioides* have focused on detecting genealogical congruence among a modest number of gene genealogies (8, 10–12). Incorporating genomic information is sorely needed to understand the magnitude of differentiation among *Paracoccidioides* pathogens. In this report, we use phylogenetics and population genetics to bridge that gap. We find that the species of *Paracoccidioides* are extensively differentiated, which suggests an advanced stage along the divergence continuum.

## RESULTS

### *Paracoccidioides* is haploid and shows little evidence for aneuploidy.

**FIG 1:**
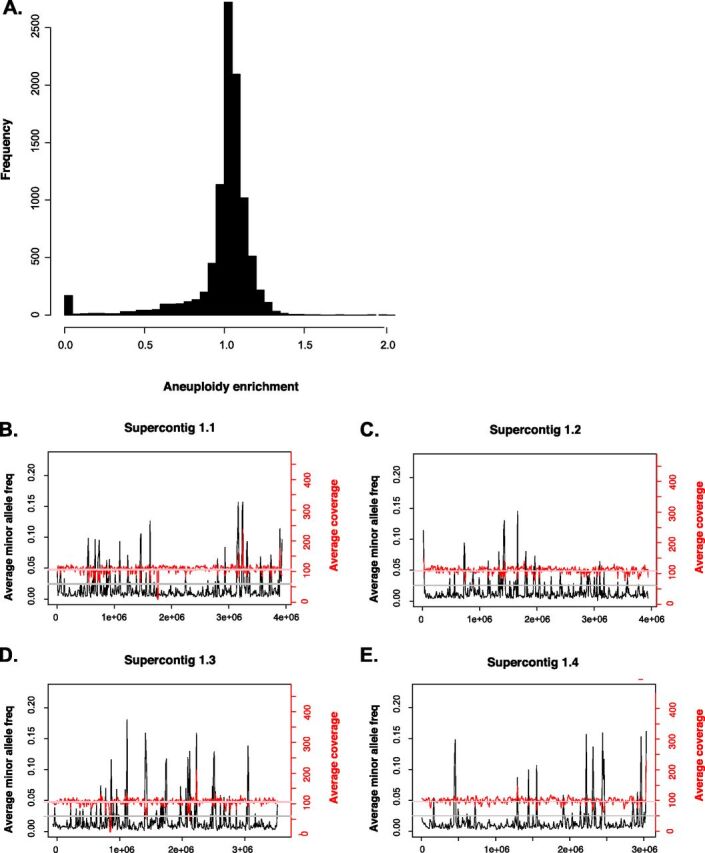
*Paracoccidioides* is haploid and shows no detectable levels of aneuploidy. (A) Mean coverage per site shows no large and consistent deviations from the expected sequencing coverage, suggesting little or no aneuploidy in *Paracoccidioides* genomes. (B) Per-site estimations of coverage (in red) show close adherence to the genome-wide mean coverage for the whole genome of the *P. lutzii* isolate EE. Per-site estimations of the extent of polymorphism across the genome suggest that no single site shows evidence of polymorphism (cutoff: minor allele frequency > 20%), suggesting that besides having no local aneuploidy, *Paracoccidioides* is haploid. The four panels on the right (B to E) show the coverage and minor allele frequency for the four largest supercontigs. Figure S2 to S5 in the supplemental material show similar plots for other *Paracoccidioides* species.

We tested two aspects regarding the ploidy of *Paracoccidioides*. First, we assessed whether the coverage in the genome was homogenous. Significant deviations from homogeneity indicate aneuploidy. Second, we tested the global and local ploidy by scoring the mean coverage and exploring for the presence of polymorphic sites. Haploid genomes, unlike higher ploidies, show no variation in each locus and should show no minor—or major—alleles. Figure 1 shows the results of this analyses. First, the global distribution of coverage is distributed evenly around 1, with a few sites getting no coverage with respect to the reference genome (Fig. 1A). Permutation tests show that this distribution is not significantly different from 1 (i.e., the expectation of homogenous ploidy along the genome; two-sample Fisher-Pitman permutation test, *P* = 0.08). When we ran similar analyses to study the mean coverage and minor allele frequency locally (i.e., in 5-kb windows) along the *Paracoccidioides* genome, we found that the expectations of haploidy were fulfilled as well (i.e., there no regions with abnormally high coverage or that were systematically polymorphic; Fig. 1B to E). Figures S2 to S5 in the supplemental material show similar analyses for all species of *Paracoccidioides*. Overall, these results confirm previous observations that *Paracoccidioides* is haploid (16); for all analyses from now on, we treat *Paracoccidioides* as such.

### All proposed species show reciprocal monophyly and strong genealogical concordance.

**FIG 2:**
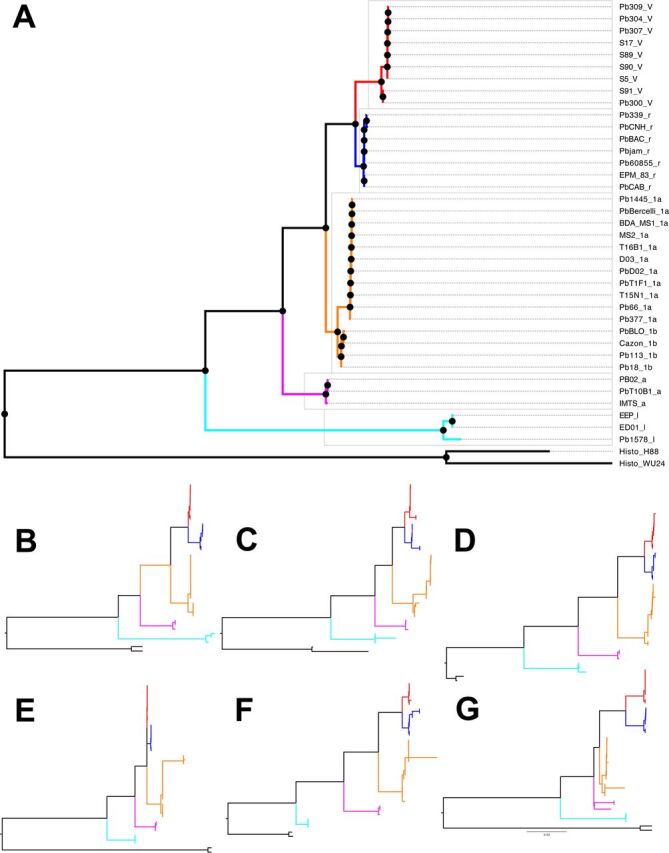
Reciprocal monophyly between *Paracoccidioides* species. (A) Maximum likelihood rooted phylogram using concatenated genome-wide loci. *Paracoccidioides venezuelensis* isolates are marked in red and have a “V” after the strain name. *Paracoccidioides restrepiensis* isolates are marked in blue and have a “r” after the strain name. Paracoccidioides brasiliensis isolates are marked in orange and have a “1a” or a “1b” after the strain name. *Paracoccidioides americana* isolates are marked in pink and have an “a” after the strain name. *Paracoccidioides lutzii* are isolates marked in cyan and have a “l” after the strain name. (B to G) Phylograms for the six largest supercontigs in the Pb18 genome show the same topology. We follow the same color scheme as that in in panel A. (B) Supercontig 1.1. (C) Supercontig 1.2. (D) Supercontig 1.3. (E) Supercontig 1.4. (F) Supercontig 1.5. (G) Supercontig 1.6. The results are consistent to those shown in Fig. 1 of Muñoz et al. (13) and Teixeira et al. (25).

We evaluated the phylogenetic relationships between all species of the *Paracoccidioides* genus using genome-wide variation and established how much of the genome supports these relationships. First, we built a maximum likelihood (ML) tree in which we used concatenated loci from the whole genome as the unit for phylogenetic analysis (this approach has important limitations [17]; see below). Figure 2A shows the resulting topology. Individual analyses of the 6 largest supercontigs yields similar topologies (Fig. 2B to G and Fig. S6 in the supplemental material), with some minor exceptions (see Fig. S6 and S7 in the supplemental material). Two results stand out from these analyses. As predicted by smaller efforts (10), all five named species of *Paracoccidioides* are reciprocally monophyletic. The tree shows that, on average, all scaffolds show an evolutionary history consistent with the clusters previously described as species, including S1a and S1b. Four out of the six supercontigs show an identical branching pattern to that of the whole-genome genealogy (Robinson-Foulds [RF] distance = 0.00). The topologies from supercontigs 2.2, 2.3, and 2.5 are slightly different from the genome-wide topology (RF distances = 24, 23, and 3.00, respectively; Fig. S7). The maximum RF distance in these trees is 70, following Bryant and Steel (18). Similarly, genome-wide phylogenetic reconstruction shows similar results to previous approaches and suggest that P. restrepiensis and P. venezuelensis are the most closely related species of the group. Paracoccidioides brasiliensis *sensu stricto* is sister to the dyad *P. venezuelensis/P. restrepiensis*, and *P. americana* is an outgroup to the other three species of the *brasiliensis* species complex.

**FIG 3:**
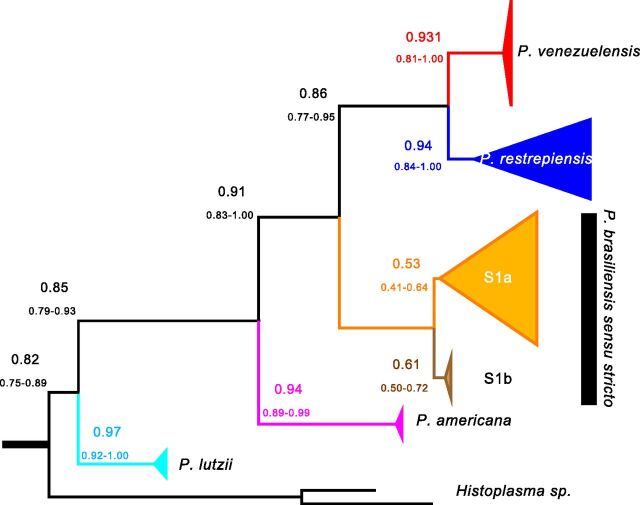
The species tree of the genus *Paracoccidioides* suggests that the majority of the genome of *Paracoccidioides* shows similar phylogenetic signal. The tree shown (built with BUCKy and a prior α of 5) is based on individual benchmarking universal single-copy ortholog (BUSCO) genes. Values above each branch show the point estimate of the concordance factor (CF) for each node; values below branches show the 95% Bayesian credibility intervals for this estimate. The majority of the genome signals reciprocal monophyly for the previously proposed species of *Paracoccidioides*.

Additionally, we calculated the concordance factors for the each of the five *Paracoccidioides* species and the putative cryptic species S1a and S1b. If speciation has proceeded to extensive genetic differentiation, then most of the genome should show the signature of reciprocal monophyly in each of the proposed species. Figure 3 shows the results of a gene genealogy concordance analysis using BUCKy. The obtained topology is identical to that produced using maximum likelihood. The concordance factors for the five proposed species is in all cases greater than 90%, which suggests that the vast majority of the genome shows concordance in the existence of the five species of *Paracoccidioides*. The only exceptions to this high level of concordance are the nodes that separate S1a and S1b, which had concordance factor (CF) values of 0.53 and 0.61, respectively. There is no metric on how high a CF value must be to elevate a group to species level (19, 20), but the genome-wide concordance of this groups is much lower than that of the other *Paracoccidioides* species. For all analyses that follow, we treat P. brasiliensis *sensu stricto* as a single species with strong population structure.

**FIG 4:**
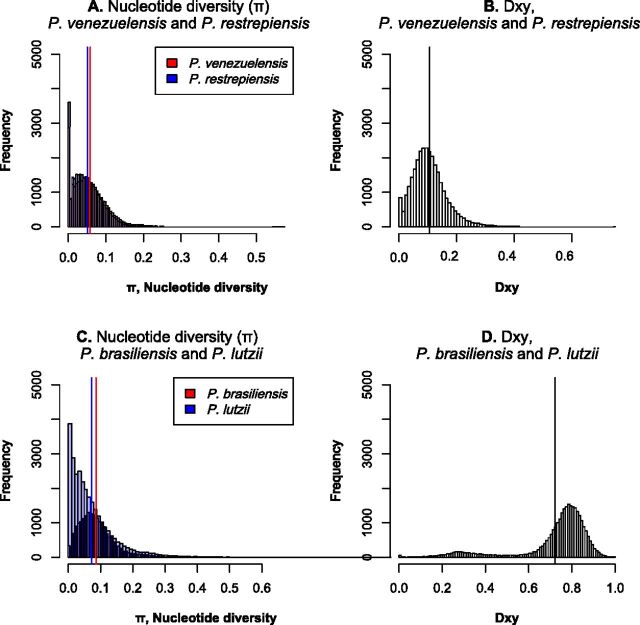
Genetic variation is partitioned across species in *Paracoccidioides*. (A and C) Distribution of pairwise divergence within four species of *Paracoccidioides*. Blue and red bars show the two mean values of π (one for each species). (B and D) Distribution of average pairwise *D*_XY_ values between two species pairs, *P. venezuelensis*/*P. restrepiensis* and *P. brasiliensis*/*P. lutzii*. Black lines show the mean value of pairwise *D*_XY_. In all cases, the distributions of intraspecific and interspecific pairwise differences are nonoverlapping (two-sample Fisher-Pitman permutation test; *P* < 1 × 10^−10^).

**FIG 5:**
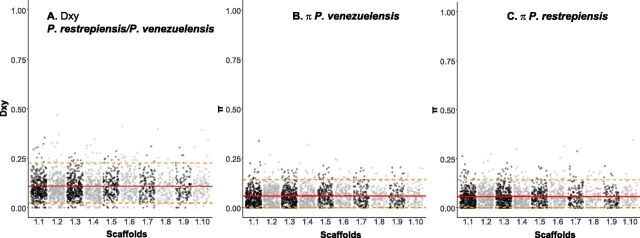
Differentiation between species of *Paracoccidioides* occurs genome wide. *D*_XY_ along the whole genome between *P. venezuelensis* and *P. restrepiensis* (left) is larger than π in either of the two species (center and left). Red solid lines show the median values; dashed orange lines show the 90th and 10th percentiles.

Next, we calculated the mean genetic distance between individuals of the same species and between pairs of individuals from different species. The expectation is that pairwise comparisons between individuals from different species should show much higher differentiation than individuals from the same species (15, 21). We found that genetic variability is partitioned among species and that in all cases the magnitude of interspecific distances is at least 2× higher than that of intraspecific distances for all species pairs in genome-wide estimations (Fig. 4 and Fig. S8 in the supplemental material). All pairwise comparisons between intraspecific and interspecific differentiation were significant (two-sample Fisher-Pitman permutation test, *P* < 1 × 10^−10^). As expected from the concordance analysis, the differentiation—measured as *D*_XY_— occurs along the whole genome (leftmost panels of Fig. 5 and Fig. S9 in the supplemental material).

The joint results from the phylogenetic analyses and the genetic distance calculations indicate that the genetic diversity within *Paracoccidioides* is partitioned across species. The five proposed species of *Paracoccidioides* fulfill the expectations of being advanced in the speciation continuum in terms of genomic divergence (22, 23). Next, we tested whether such divergence is accompanied by a reduction in the amount of gene flow between species.

### Low rates of detectable gene exchange between species.

If speciation has proceeded to advanced stages, as seems to be the case for the species in *Paracoccidioides*, the magnitude of gene exchange between species should be limited. To detect potential alleles that have crosses species boundaries, we used two different methods, *D*-statistics and Int-HMM, a method that detects haplotypes likely to have crossed species boundaries. We describe the results for each species pair as follows.

**(i) *Paracoccidioides lutzii* and the species from the *brasiliensis* complex.** Using *Int-HMM*, we found no evidence of introgression in any of these species pairs or in any reciprocal direction. If hybridization and admixture has occurred between P. lutzii and the other *Paracoccidioides* species, it has left no trace in the genomes of any of the involved species.

### (ii) *Paracoccidioides americana* and the rest of the species from the *brasiliensis* complex.

*D* and *f_D_*, two metrics to detect introgression from phylogenetic trees (see Materials and Methods for details), suggest that P. americana has donated more genetic material to P. brasiliensis and P. venezuelensis than it has donated to P. restrepiensis. In all cases the proportion of introgression is small (i.e., *f_D_* is lower than 0.04; Table 1). We followed up with Ancestry-HMM and found no evidence of large haplotypes (over 500 bp) between *P. americana* and the other species from the *brasiliensis* complex. This result suggest that introgressions are small and are probably old or strongly selected against. Regardless of the explanation for this low proportion of admixture, the joint results indicate that the magnitude of gene exchange between *P. americana* and the other species of the *brasiliensis* species complex is low.

**TABLE 1:** *D* and *f_D_* values, two metrics to detect introgression for species tetrads involving *P. americana* as a donor^*a*^

Species tetrad (P1-P2-P3)^*b*^	*D*	*f_D_*
*P. restrepiensis-P. venezuelensis-P. americana*	0.415	0.040
*P. restrepiensis-P. brasiliensis-P. americana*	0.304	0.019
*P. brasiliensis-P. venezuelensis-P. americana*	0.011	4.954 × 10^−3^

a A positive *D* value means more introgression between P3 (*P. americana*) and P2 (the second species listed) than between P3 and P1 (the first species in the list); a negative *D* value means introgression between P3 and P1.

b In all cases, *P. lutzii* is the outgroup.

**(iii) Paracoccidioides brasiliensis/*P. restrepiensis* and *P. brasiliensis*/*P. venezuelensis*.** Unlike pairwise comparisons involving the more divergent species pairs, we found evidence of limited gene exchange using both methods in these two species pairs. The only tetrad that fulfilled the requirements for the calculation of *D* was [(((*venezuelensis*, *restrepiensis*), *brasiliensis*), *lutzii*)]. The evidence of gene flow in this case was strong and showed that introgression between *P. brasiliensis* and *P. venezuelensis* is more common than between *P. brasiliensis* and *P. restrepiensis* (*D* = 0.331, *P* < 0.001; degrees of freedom [df] = 0.078). This amount of introgression is higher than that observed between *P. americana* and other species in the *brasiliensis* species group.

**FIG 6:**
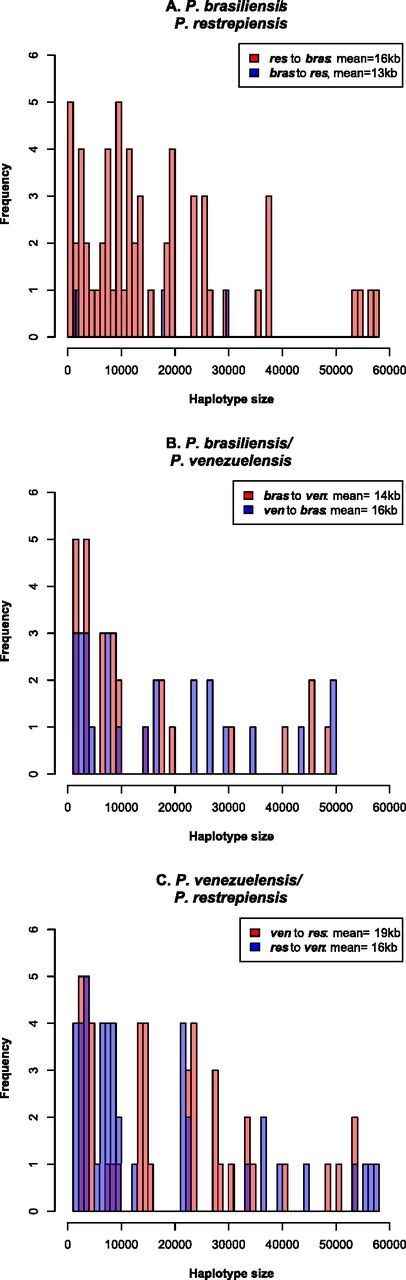
Distributions of the size of introgressed haplotypes in three species pairs of *Paracoccidioides* identified using Int‐HMM. The Int‐HMM algorithm was run on each individual separately, and the distribution of haplotype sizes was computed for each of the two directions of introgression. (A) Size distribution of introgressed haplotypes between *P. brasiliensis sensu stricto* and *P. restrepiensis*. (B) Size distribution of introgressed haplotypes between *P. brasiliensis sensu stricto* and *P. venezuelensis*. (C) Size distribution of introgressed haplotypes between *P. venezuelensis* and *P. restrepiensis*. Even though there are large introgressions (over 50 kb) in most crosses, the majority of the introgressions are small.

**FIG 7:**
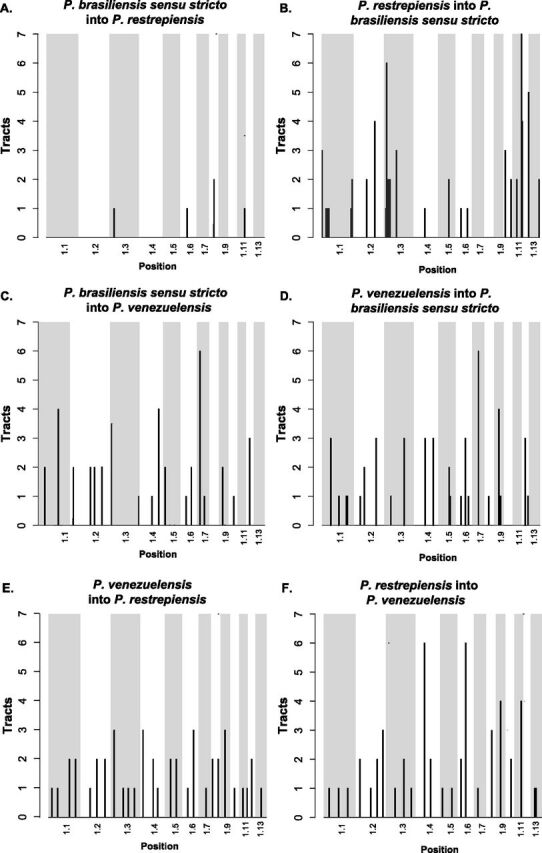
Location of the introgression tracts between species of *Paracoccidioides*. Each pair of panels shows the two reciprocal directions of introgression for a species pair. (A and B) *P. restrepiensis* and *P. brasiliensis sensu stricto*. (C and D) *P. venezuelensis* and *P. brasiliensis sensu stricto*. (E and F) *P. restrepiensis* and *venezuelensis*. The two directions show differences in the amount of introgression, hence the differences in the scale of the *y* axis. We plotted the largest 13 supercontigs of the genome. Histograms show the number of introgression tracts found in 500-kb windows.

Next, we used Int-HMM to study the characteristics of the introgressed haplotypes. Table 2 shows the percentage of genome that has crossed species boundaries in each sequenced genome. We found no overlap of the introgression regions between reciprocal directions or species pairs. In *P. brasiliensis/P. restrepiensis*, introgression mean length did not differ between reciprocal directions (Welch two-sample *t* test data; *t* = −0.857, df = 4.135, *P* = 0.438) and in both cases was ∼15 kb, suggesting similar times of admixture or similar selection against introgression (Fig. 6A). We found a similar pattern in *P. brasiliensis/P. venezuelensis.* The haplotype length did not differ between reciprocal directions (Welch two-sample *t* tests; *P. brasiliensis-restrepiensis*: *t* = 0.449, df = 3.456, *P* = 0.680; *P. brasiliensis-P. venezuelensis*: *t* = 0.536, df = 50.795, *P* = 0.594) and in both cases was ∼15 kb (Fig. 6B). In all cases, introgressions occur at low frequency (i.e., present in a single isolate per species) and are mostly located in intergenic regions (Table 3). Figure 7A to D shows the location of introgressed haplotypes in the two directions of the cross. Introgressions were distributed along most supercontigs, suggesting that most supercontigs are equally permissive—or refractory—to introgression (Fig. 7).

**TABLE 2:** Percentage of the genome that shows introgression in each sequenced *P. restrepiensis*, *P. venezuelensis*, and *P. brasiliensis* isolate

Species^*a*^	Isolate	% of genome showing introgression from^*b*^:		
		*P. restrepiensis*	*P. brasiliensis*	*P. venezuelensis*
*P. restrepiensis*	EPM_83	NA	0	1.20
*P. restrepiensis*	Pb60855	NA	0.18	1.14
*P. restrepiensis*	PbBAC	NA	0	0
*P. restrepiensis*	PbCAB	NA	0	0
*P. restrepiensis*	PbCNH	NA	8.10 × 10^−3^	0
*P. restrepiensis*	PbJAM	NA	0.07	0.14
*P. brasiliensis sensu stricto*	MS1	0.61	NA	1.51
*P. brasiliensis sensu stricto*	DO3	0.13	NA	2.33
*P. brasiliensis sensu stricto*	Pb1445	0.87	NA	1.14
*P. brasiliensis sensu stricto*	Pb377	0.0	NA	0
*P. brasiliensis sensu stricto*	PbBercelli	0.66	NA	0
*P. brasiliensis sensu stricto*	PbD02	0.10	NA	0.95
*P. brasiliensis sensu stricto*	PbT1F1	0.14	NA	1.21
*P. brasiliensis sensu stricto*	T15N1	0.05	NA	0.78
*P. brasiliensis sensu stricto*	T16B1	0.26	NA	0.54
*P. brasiliensis sensu stricto*	MS2	0.67	NA	0.06
*P. brasiliensis sensu stricto*	Pb_66	0.29	NA	0.02
*P. venezuelensis*	Pb309	1.20	0.87	NA
*P. venezuelensis*	Pb304	0.45	1.10	NA
*P. venezuelensis*	Pb307	0	0	NA
*P. venezuelensis*	PbS89305	1.41	0.95	NA
*P. venezuelensis*	PbS90384	0.55	0.05	NA
*P. venezuelensis*	PbS5387	0.14	0.13	NA
*P. venezuelensis*	PbS91444	0.13	0.04	NA
*P. venezuelensis*	Pb300	0	0.11	NA

a Isolates from *P. lutzii* and *P. americana* show no evidence of large (over 500 bp) introgressed haplotypes and are not listed.

b NA, not applicable.

**TABLE 3:** Introgressions between *P. restrepiensis* and *P. brasiliensis sensu stricto* are mostly found in intergenic regions

Direction	Sequence type^*a*^	Length (kb)	Introgressed %^*b*^	Genomic %^*c*^	Enrichment^*d*^
*P. restrepiensis* into *P. brasiliensis*	10-kb inter	9.78	42.8565	23.990	1.786
*P. restrepiensis* into *P. brasiliensis*	2-kb upstream inter	6.28	27.4918	29.759	0.9245
*P. restrepiensis* into *P. brasiliensis*	3′ UTR	0.03	0.1227	1.817	0.068
*P. restrepiensis* into *P. brasiliensis*	5′ UTR	0	0	1.019	0
*P. restrepiensis* into *P. brasiliensis*	CDS	5.92	25.9365	44.598	0.582
*P. restrepiensis* into *P. brasiliensis*	Intergenic	0	0	1.904	0
*P. restrepiensis* into *P. brasiliensis*	Intron	0.82	3.5926	12.225	0.294
*P. brasiliensis* into *P. restrepiensis*	10-kb inter	980.29	26.1924	23.990	1.092
*P. brasiliensis* into *P. restrepiensis*	2-kb upstream inter	1,436.81	38.39	29.759	1.290
*P. brasiliensis* into *P. restrepiensis*	3′ UTR	24.31	0.6495	1.817	0.358
*P. brasiliensis* into *P. restrepiensis*	5′ UTR	10.12	0.2704	1.019	0.266
*P. brasiliensis* into *P. restrepiensis*	CDS	1,071.78	28.637	44.598	0.642
*P. brasiliensis* into *P. restrepiensis*	Intergenic	113.07	3.0212	1.904	1.587
*P. brasiliensis* into *P. restrepiensis*	Intron	106.27	2.8395	12.225	0.232

a To study whether any particular type of sequence was over- or underrepresented, we partitioned the genome by sequence type with each region being assigned to one of the following eight sequence types: coding sequence (CDS), exon, 5′ untranslated region (UTR), 3′ UTR, intron, 2-kb upstream inter (intergenic sequence 2 kb upstream of a gene), 10-kb inter (intergenic sequence within 10 kb of a gene), and intergenic (intergenic sequence more than 10 kb from a gene).

b The introgressed percentage is the percentage of introgressions overlapping a given sequence type for that direction.

c The genomic percentage is the percentage of the genome represented by a given sequence type.

d Enrichment = (introgressed percentage)/(genomic percentage).

**(iv) *P. venezuelensis*/*P. restrepiensis*.** Finally, we studied the most recently diverged species pair in *Paracoccidioides* using Int-HMM. We found no overlap in the location of haplotypes between the two reciprocal directions or with any of the other dyads of *Paracoccidioides*. There was no difference in the genome proportion introgressed per individual between reciprocal directions (Welch two-sample *t* test; *t* = 0.11, df = 9.95, *P* = 0.91). The mean haplotype length did not differ between reciprocal directions (Welch two-sample *t* test; *t* = 0.741, df = 85.12, *P* = 0.461) and in both cases was ∼16 kb (Fig. 6C). As in the case for the other *Paracoccidioides* dyads, introgressions were at a low frequency and were largely in intergenic regions (Table 3). Notably, we found similar amounts of introgression in this pair as we found in the more divergent pairs (Welch two-sample *t* test; *P. venezuelensis*/*P. restrepiensis* versus *P. venezuelensis*/*P. brasiliensis sensu stricto*, *t* = 1.205, df = 31.962, *P* = 0.237; *P. venezuelensis*/*P. restrepiensis* versus *P. restrepiensis*/*P. brasiliensis sensu stricto*, *t* = −1.372, df = 21.731, *P* = 0.184). Figure 6C shows the haplotype size frequency distribution of introgressions in the two reciprocal directions. Introgressions were distributed along the whole genome and did not follow a particular clustering pattern (Fig. 7E and F).

## DISCUSSION

Our study uses genomic data to confirm previous observations that five species of *Paracoccidioides* (i) are all haploid, and (ii) are genetically differentiated. We also present results that suggest that the genomes of these species show strong levels of genealogical concordance genome wide and rarely exchange genes. The species of *Paracoccidioides* show considerable divergence and reciprocal monophyly which in turn suggest these five species are at an advanced stage on the speciation continuum (22–24).

Our analyses of the magnitude of gene flow between species confirm that despite extensive geographic overlap, the *Paracoccidioides* species rarely exchange genes. In the more divergent pairs, those of the *brasiliensis* complex and *P. lutzii*, we found no evidence of introgression. This is consistent with the high levels of divergence between *P. lutzii* and the species from the *brasiliensis* complex, which have been hypothesized to be over 30 million years apart (10, 12). We observed a similar—but not identical— pattern between P. americana and the other species of the *brasiliensis* complex (*P. brasiliensis*, *P. restrepiensis*, and *P. venezuelensis*). Potential introgressions between these species are rare and of very small size, making them potentially indistinguishable from incomplete lineage sorting, as there was no evidence of gene exchange in any of the pairs. This paucity of gene exchange is not caused by lack of contact. Paracoccidioides brasiliensis, *P. lutzii*, and *P. americana* coexist in Brazil and have even been found in the same host (8, 25). *Paracoccidioides venezuelensis* and *P. americana* share their geographic range in Venezuela as well. This extensive geographic overlap suggests that there is ample opportunity for gene exchange, but it does not occur.

We do find evidence of moderate gene exchange in the triad *P. brasiliensis-P. restrepiensis-P. venezuelensis*. These low levels of gene exchange are consistent with advanced divergence among *Paracoccidioides* species. Our scans for gene exchange pose two additional questions. First, the rate of gene exchange is symmetrical in two *Paracoccidioides* species pairs, *P. brasiliensis/P. venezuelensis* and *P. venezuelensis/P restrepiensis*. The third species pair, *P. brasiliensis sensu stricto*/*P. restrepiensis*, shows strongly asymmetric introgression that is mostly found in intergenic regions. The reasons behind this pattern remain unknown. We formulate two possibilities. First, the direction of migration might be asymmetric between these two species. If *P. brasiliensis* migrants come into the range of *P. restrepiensis* more often than the reciprocal type of migration, then *P. brasiliensis* alleles should be found more frequently in the *P. restrepiensis* background than the reciprocal. A second possibility is that the *P. restrepiensis* background is less permissive of introgression because the introgressed alleles might have more deleterious effects. Since *P. restrepiensis* has a much smaller effective population size (8), variants that might ameliorate the potentially deleterious effect of introgressed alleles should be rarer. On the other hand, small populations might harbor fewer deleterious mutations (26). The rates of migration, hybridization—or even intraspecific recombination— and of potential hybrid incompatibilities are unknown in *Paracoccidioides*, and we cannot disentangle these possibilities.

A second intriguing pattern is that *P. venezuelensis* and *P. restrepiensis* show a similar level of introgression to those of the other species pairs. As divergence increases, so should the number of incompatibilities (27–29), which in turn should reduce the proportion of genome that can flow from one species to the other (30, 31). Since the *P. venezuelensis*/*P. restrepiensis* dyad is more closely related than other species pairs within the *brasiliensis* complex, we expected a higher level of gene exchange. Our results do not support this hypothesis. Even though the precise reasons for this pattern remain unexplored, the role of geography might be of particular importance. The ranges of *P. venezuelensis* and *P. restrepiensis* are contiguous but have not been reported to overlap. This differs from all other species pairs in *Paracoccidioides*, which show some degree of geographic overlap (25). A precise assessment of the range and opportunity for hybridization will be crucial to establish the genetic, environmental, and demographic factors that govern the patterns of introgression in *Paracoccidioides*.

The identification of species boundaries and introgression in fungal pathogens has human health-related implications. *Paracoccidioides lutzii* and *P. brasiliensis sensu stricto* show differences in the immunological response they elicit (14, 32), the strength of the disease they cause (14), and in traits involved in diagnostic tools (33–35). Introgression, then, can be a vehicle to transfer virulence factors and antifungal resistance in *Paracoccidioides*. Gene exchange can also be a source of variation in other fungal pathogens (36–38). A systematic survey to characterize the virulence and resistance of differentiated species across their whole geographic range could reveal the extent to which diversification of the ethological agents of PCM has also led to divergence in virulence strategies. The combination of phenotypic studies and population genetics can also reveal whether gene exchange plays a role on the transfer of virulence factors and antifungal resistance strategies.

Our results are in line with those of other studies that show that species boundaries in fungi are semipermeable (15, 39, 40) and that introgression might not be rare. On the other hand, they also reveal that introgression is not an unavoidable outcome of secondary contact. Geographic overlap is not synonymous with hybridization, and in cases of diverged species (such as *P. lutzii* and the species of the *brasiliensis* species complex), hybrids might not occur even when species share a close geographic range. Hybrids might also be sterile or inviable (41). Genome factors such as the amount of divergence between hybridizing species (30) and the landscape of recombination (42, 43) affect whether an introgression persists after hybridization. The different levels of divergence and the ample opportunity for hybridization among *Paracoccidioides* species provide for a system to test the relative importance of genomic factors in determining the amount of introgression occurs in nature.

## MATERIALS AND METHODS

### Public data.

All of the data used here have been previously published. The SRA numbers are listed in Table S1 in the supplemental material. To root our trees (see below), we obtained sequencing reads from two species of Histoplasma: Histoplasma capsulatum *sensu stricto* and Histoplasma mississippiense (SRA BioProject accession number PRJNA416769) (44). These species are among some of the closest relatives of *Paracoccidioides* (45).

10.1128/mBio.01999-20.1 TABLE S1 SRA numbers of the genome sequences used in this study. Download Table S1, DOCX file, 0.02 MB. Copyright © 2020 Mavengere et al. 2020 Mavengere et al. This content is distributed under the terms of the Creative Commons Attribution 4.0 International license.

### Read mapping and variant calling.

Reads were mapped to the Paracoccidioides brasiliensis strain Pb18 genome (BioProject accession number PRJNA28733 and BioSample accession number SAMN02953720), currently assembled into 57 supercontigs, using Burrows-Wheeler Aligner (BWA) version 0.7.12 (46). BAM files were then merged using SAMtools version 0.1.19. Indels were identified and reads locally remapped in the merged BAM files using the GATK version 3.2-2 RealignerTargetCreator and IndelRealigner functions (47, 48). Subsequently, single-nucleotide polymorphisms (SNPs) were called using the GATK UnifiedGenotyper function with the parameter “het” set to 0.01 and all others left as default. The following filters were applied to the resulting VCF file: QD = 2.0, FS_filter = 60.0, MQ_filter = 30.0, MQ_Rank_Sum_filter = −12.5, and Read_Pos_Rank_Sum_filter = −8.0. Sites were excluded if the coverage was less than 5 or greater than the 99th quantile of the genomic coverage distribution for the given line or if the SNP failed to pass one of the GATK filters.

### Ploidy estimation.

To detect admixture between species of *Paracoccidioides*, we used Int-HMM (49), an algorithm to detect introgression that requires information on the ploidy of an individual (i.e., it can be run to detect introgression in diploid or haploid organisms; see below). We used genome-wide data to determine the most likely ploidy of the *Paracoccidioides* isolates. We used Illumina short reads from the five species of *Paracoccidioides* (described above) to do two ploidy tests. First, we plotted the per-site sequencing coverage. In cases in which there is partial aneuploidy in the form of chromosomal duplications, there will be a bimodal distribution. In cases where the genome does not harbor aneuploid regions, there will be a single mode in the distribution. To compare the observed distribution of per-site coverage with the null hypothesis of uniform sequencing coverage, we used a two-sample Fisher-Pitman permutation test (function *oneway_test*, library *coin* [50]). We used the “hist” function (library *graphics* [51]) in R to plot the distribution of the per site coverage and of allele frequencies per site across the whole genome for each strain.

Next, we studied the ploidy of *Paracoccidioides* at a local level. We used the same two metrics described above. Sites with the ploidy of the rest of the genome should show a mean per-window coverage. Once-duplicated segments (either as copy number variation or as changes in ploidy) should have twice the coverage of the genome average. We thus calculated the coverage and mean minor allele frequency for each 5-kb window in the genome to assess whether there were segments of the genome with evidence for changes in ploidy.

### Phylogenetic reconstructions.

Our goal was to determine whether the species from *Paracoccidioides* were reciprocally monophyletic and thus satisfy the requirements to be considered phylogenetic species. We followed a phylogenetic species concept (15, 52, 53) to recognize species, defining species as genetic clusters that are reciprocally monophyletic and for which there was genealogical concordance across genome-wide unlinked loci. We used two parallel approaches: (i) maximum likelihood trees at the genome and at the supercontig level, and (ii) Bayesian concordance analysis of the genealogies of orthologous genes. We describe each of these two approaches as follows.

**(i) Maximum likelihood phylogenetics.** To determine whether the proposed *Paracoccidioides* species were monophyletic, we first used maximum likelihood phylogenetics. Reciprocal monophyly is a trademark of speciation (15, 22); as divergence accrues, the likelihood of reciprocal monophyly across the whole genome increases for two reasons. First, as divergence increases, the likelihood of incomplete lineage sorting decreases (54, 55). Likewise, in diverged lineages, the magnitude of retained introgression is lower even in cases where hybridization might occur frequently (30, 31, 49). Since recombination in *Paracoccidioides* occurs but seems to be rare, and there is a high level of linkage disequilibrium across the genome (see Results), we studied each supercontig as an unlinked locus. Since mitochondrial DNA (mtDNA) shows evidence of interspecific gene transfer (10, 56), we focused only on nuclear genomes. We obtained whole supercontig sequences for each individual from the VCF file using the FastaAlternateReferenceMaker tool in GATK, realigned them using Mafft version 7 (57), and used them to build maximum likelihood (ML) trees using RAxML version 8.2.9 (58). We inferred individual trees for each of the largest six supercontigs, which encompass 62% of the genome. We also generated a genome-wide tree of a concatenated alignment of all supercontigs. Analyses were run under the GTR + Γ model, with 1,000 bootstrap pseudoreplicates to assess support for each node. The genome-wide analysis was partitioned by supercontig, with each partition having its own set of GTR + Γ model parameters. All trees were rooted using *Histoplasma mississippiense and* H. capsulatum *sensu stricto* (44). To determine the extent of congruence among supercontig trees and the whole-genome tree, we used the Robinson-Foulds (RF) (or symmetric difference [59]) distance (function *treedist*, library *phangorn* [60]), which counts how many partitions are in one tree but not the other. Even though this is a simplistic approach that does not take into account branch length information (18, 61, 62), it reveals whether there are large-scale levels of incongruency in the topology. We also plotted the concatenated tree topology and superimposed the trees from each supercontig using the R function *compare.chronograms* (library *phytools* [63]) and the nodes that are present in the whole-genome sequence tree but not in the supercontig genealogies using the function *comparePhylo* (library *ape* [64]).

**(ii) Bayesian concordance analyses.** In cases where speciation has occurred and genetic divergence has accrued, the phylogenetic signal across the whole genome should be congruent across loci. We measured the genome-wide genealogical concordance at a finer scale using a Bayesian concordance analysis (BCA). First, we identified orthologous genes using the BUSCO annotation pipeline (65, 66), which encompasses 1,316 benchmarking universal single-copy orthologs. These groups of genes have been curated in 25 different species of ascomycetous fungi (65, 66). Next, we used MrBayes version 3.2.6 (67, 68) to generate posterior tree distributions for each single gene. We summarized the gene trees using the command *mbsum* in the BUCKy program (69–71) with a burn-in of 1,000 trees. We then fed individual gene genealogies to BUCKy version 1.4.2, with four independent runs and four Markov chain Monte Carlo (MCMC) chains, each with 10 million generations with a burn-in period of 100,000. Five values of the α parameter (0.1, 0.5, 1, 5, and 10) were tested, which correspond to the prior probability distribution for the number of distinct gene trees (70). The level of support for each node is expressed as a concordance factor (CF), which ranges between 0 (no concordance between genealogies) and 1 (complete concordance). This approach allowed us to infer the phylogenetic relationships between putative species, while estimating the genome-wide genealogical support for their monophyly.

### Genetic distance.

To further assess the extent of genetic differentiation between phylogenetic species, we used the metric $\pi_{\text{INTER}}$, the average number of nucleotide differences between one sequence randomly chosen from a population and another sequence randomly chosen from a second population or species. *D*_XY_, or $\pi_{\text{INTER}}$, followed the form:

$$\pi_{\text{INTER ij}}=\;\frac{\text{pairwise differences between individuals }i\text{ and }j\;}{\text{sequence length}}$$

Mean $\pi_{\text{INTER}}$ was the mean of all pairwise comparisons between individuals from two species. We calculated 20 mean $\pi_{\text{INTER}}$ values. We also calculated $\pi_{\text{INTRA}}$, the average pairwise genetic distance between individuals of the same species. $\pi_{\text{INTRA}}$ followed the same form as $\pi_{\text{INTER}}$, but instead of calculating the average number of differences between species, it calculates the average number of differences between two randomly selected individuals of the same species. Mean $\pi_{\text{INTRA}}$ was the mean within-species value for each of the five species. We used Python for all calculations.

In cases were speciation is complete, $\pi_{\text{INTER}}$ is expected to be much larger than $\pi_{\text{INTRA}}$. For each species pair, $\pi_{\text{INTRA}}$ can take two values (i.e., from each of the two species), so for the pairwise comparisons $\pi_{\text{INTRA}}$ is the pooled set of the two intraspecific distances. To compare the values of $\pi_{\text{INTER}}$ and $\pi_{\text{INTRA}}$ for each species pair (10 pairwise comparisons), we used two-sample Fisher-Pitman permutation tests as implemented by the function *oneway_test* in the *coin* library in R (9,999 Monte Carlo resamplings) (50). We also calculated $\pi_{\text{INTER}}$ for each of the 10 pairwise comparisons and $\pi_{\text{INTRA}}$ for the five *Paracoccidioides* species for each of the largest 6 supercontigs.

### Gene exchange between species of *Paracoccidioides*.

Previous work based on coding and microsatellite data suggested the possibility of gene exchange between *Paracoccidioides* species (10). However, although microsatellite makers have the potential to reveal genealogical relationships between very closely related individuals, they are also prone to homoplasy, as they mutate quickly and their identity might not be caused by descent (72–74). To address the possibility of gene exchange with better resolution, we used whole-genome data and two complementary methods, *D*-statistics and Int-HMM.

**(i) *D*-statistics.** First, we calculated the excess of variants shared between potentially admixed species using *D*-statistics (75–78). *D* is a metric to detect introgression from phylogenetic trees. The metric requires a four-taxon topology [(((P1, P2), P3), O)]. The allele in the outgroup (O) is labeled A, while the derived allele in the ingroup is labeled B. *D* compares the occurrence of two discordant site patterns, ABBA and BABA, representing sites in which an allele is derived in P3 relative to O and is derived in one but not both of the sister lineages P1 and P2. These discordant patterns are most likely to arise if introgression occurs between P3 and either P2 or P1, in which case one site pattern will occur more frequently than the other. A positive *D* value means introgression between P3 and P2; a negative *D* value means introgression between P3 and P1. Due to the need for a sorted topology, we focused on four species tetrads where [(((P1, P2), P3), O)] were as follows: [(((*venezuelensis*, *restrepiensis*), *americana*), *lutzii*)], [(((*venezuelensis*, *restrepiensis*), *brasiliensis*), *lutzii*)], [(((*venezuelensis*, *brasiliensis*), *americana*), *lutzii*)], [(((*restrepiensis*, *brasiliensis*), *americana*), *lutzii*)].

For each species pair, we measured the standard deviation of *D* from 1,000 bootstrap replicates. The observed genome-wide *D* was converted to a *Z*‐score measuring the number of standard deviations it deviates from 0, and significance was assessed from a *P* value using an α of 0.01 as a cutoff after Holm-Bonferroni correction for multiple testing. We also calculated a variation of *D*, *f_D_*, which estimates the proportion of admixture by dividing the observed difference between the ABBA and BABA counts to the expected difference when the entire genome is introgressed. Besides the genome-wide average of *D* and *f_D_*, we calculated both metrics for 5-kb windows along the genome. We used DSuite for all calculations (79), and used the allele frequencies within each species, as recommended in reference 77.

**(ii) Identification of introgressed haplotypes with Ancestry-HMM.** We used a hidden Markov model (HMM) able to detect introgression in diploids and haploids (i.e., Int-HMM [36, 37, 49]) to identify introgressed regions between all pairs of *Paracoccidioides* species. The HMM identifies introgressions between a pair of diverged populations or species, a donor and a recipient (i.e., the admixed individual), by inferring the ancestry of every SNP in the genome. It then identifies a consecutive group of SNPs from the donor in the recipient background. Donor SNPs were selected such that they were monomorphic in the donor species and the allele frequency differences between the two species was greater than or equal to 30%. We also required that every individual in the donor species and at least one individual in the recipient species had a called genotype. Transition and emission probabilities of the HMM have been described elsewhere (36, 37).

**(iii) Identifying introgression tracts.** Int-HMM determined the most probable genotype for each marker in each individual. We defined tracts as contiguous markers with the same genotype (species 1 or species 2). Introgressed SNPs are defined as those within a tract where the HMM probability for an introgression state (*d*) (i.e., originating from the donor) was ≥50%. In cases where we identified a region of *d* with at least 10 introgressed SNP markers flanked on one side by a small tract (under 10 SNPs) from the recipient that in turn was flanked by a single larger tract that was completely *d*, the two introgressed regions were merged and consolidated into a single tract. We did four consecutive rounds of filtering to allow identification of larger introgressed tracts that were broken up by small sections of the recipient species. These broken regions might be caused by gene conversion, double recombination events, or sequencing error (Fig. S1 in the supplemental material shows an example).

10.1128/mBio.01999-20.2 FIG S1 Example of an introgression from *P. restrepiensis* to *P. brasiliensis* defined by Int-HMM. Blue bars represent *P. brasiliensis* ancestry; red bars represent *P. restrepiensis* ancestry. Light red bars represent potential *P. restrepiensis* introgressions with low support (i.e., few markers). Download FIG S1, PDF file, 0.1 MB. Copyright © 2020 Mavengere et al. 2020 Mavengere et al. This content is distributed under the terms of the Creative Commons Attribution 4.0 International license.

10.1128/mBio.01999-20.3 FIG S2 *Paracoccidioides americana* is haploid and shows no detectable levels of aneuploidy. Coverage and minor allele frequency plots for the largest 16 supercontigs. Per-site estimations of coverage (in red) show close adherence to the genome-wide mean coverage for the whole genome. Per-site estimations of the extent of polymorphism across the genome suggest that no single site shows evidence of polymorphism (cutoff: minor allele frequency > 20%), suggesting that besides having no local aneuploidy, *Paracoccidioides* is haploid. The plot shows results for isolate T10B1; plots for other lines can be found in the Dryad package (https://doi.org/10.5061/dryad.w3r2280pj). Download FIG S2, PDF file, 0.1 MB. Copyright © 2020 Mavengere et al. 2020 Mavengere et al. This content is distributed under the terms of the Creative Commons Attribution 4.0 International license.

10.1128/mBio.01999-20.4 FIG S3 Paracoccidioides brasiliensis is haploid and shows no detectable levels of aneuploidy. Coverage and minor allele frequency plots for the largest 16 supercontigs. Per-site estimations of coverage (in red) show close adherence to the genome-wide mean coverage for the whole genome. Per-site estimations of the extent of polymorphism across the genome suggest that no single site shows evidence of polymorphism (cutoff: minor allele frequency > 20%), suggesting that besides having no local aneuploidy, *Paracoccidioides* is haploid. The plot shows results for isolate T1F1; plots for other lines can be found in the Dryad package (https://doi.org/10.5061/dryad.w3r2280pj). Download FIG S3, PDF file, 0.1 MB. Copyright © 2020 Mavengere et al. 2020 Mavengere et al. This content is distributed under the terms of the Creative Commons Attribution 4.0 International license.

10.1128/mBio.01999-20.5 FIG S4 *Paracoccidioides restrepiensis* is haploid and shows no detectable levels of aneuploidy. Coverage and minor allele frequency plots for the largest 16 supercontigs. Per-site estimations of coverage (in red) show close adherence to the genome-wide mean coverage for the whole genome. Per-site estimations of the extent of polymorphism across the genome suggest that no single site shows evidence of polymorphism (cutoff: minor allele frequency > 20%), suggesting that besides having no local aneuploidy, *Paracoccidioides* is haploid. The plot shows results for isolate EPM83; plots for other lines can be found in the Dryad package (https://doi.org/10.5061/dryad.w3r2280pj). Download FIG S4, PDF file, 0.1 MB. Copyright © 2020 Mavengere et al. 2020 Mavengere et al. This content is distributed under the terms of the Creative Commons Attribution 4.0 International license.

10.1128/mBio.01999-20.6 FIG S5 *Paracoccidioides venezuelensis* is haploid and shows no detectable levels of aneuploidy. Coverage and minor allele frequency plots for the largest 16 supercontigs. Per-site estimations of coverage (in red) show close adherence to the genome-wide mean coverage for the whole genome. Per-site estimations of the extent of polymorphism across the genome suggest that no single site shows evidence of polymorphism (cutoff: minor allele frequency > 20%), suggesting that besides having no local aneuploidy, *Paracoccidioides* is haploid. The plot shows results for isolate Pb300; plots for other lines can be found in the Dryad package (https://doi.org/10.5061/dryad.w3r2280pj). Download FIG S5, PDF file, 0.1 MB. Copyright © 2020 Mavengere et al. 2020 Mavengere et al. This content is distributed under the terms of the Creative Commons Attribution 4.0 International license.

10.1128/mBio.01999-20.7 FIG S6 Graphic representation of tree concordance between the whole-genome topology and the genealogy produced from each supercontig. Arrows show differences in branch length between trees built with the whole genome (light red) and data from each supercontig (light blue). The plots were made using *compare.chronograms* (library *phytools* [63]). Download FIG S6, PDF file, 0.2 MB. Copyright © 2020 Mavengere et al. 2020 Mavengere et al. This content is distributed under the terms of the Creative Commons Attribution 4.0 International license.

10.1128/mBio.01999-20.8 FIG S7 Differences in topology between the trees from the whole genome and those from each supercontig. We only show the results for the tree supercontigs that showed differences with the whole-genome tree, as follows: (A) supercontig 2.2, (B) supercontig 2.3, and (C) supercontig 2.5. Blue circles show clades absent in the supercontig tree but present in the whole-genome tree. Download FIG S7, PDF file, 0.1 MB. Copyright © 2020 Mavengere et al. 2020 Mavengere et al. This content is distributed under the terms of the Creative Commons Attribution 4.0 International license.

10.1128/mBio.01999-20.9 FIG S8 π and **D**_XY_ calculations for pairwise comparisons that included *P. lutzii*. Color scheme and arrangement are similar to those in Fig. 4. Note that panels A and B are identical to panels C and D in Fig. 4. Download FIG S8, PDF file, 0.3 MB. Copyright © 2020 Mavengere et al. 2020 Mavengere et al. This content is distributed under the terms of the Creative Commons Attribution 4.0 International license.

10.1128/mBio.01999-20.10 FIG S9 Differentiation between species of *Paracoccidioides* occurs genome wide. **D**_XY_ along the whole genome between *P. brasiliensis* and *P. restrepiensis* (left) is larger than π in either of the two species (center and left). Red solid lines show the median values; dashed orange lines show the 90th and 10th percentiles. Download FIG S9, PDF file, 0.8 MB. Copyright © 2020 Mavengere et al. 2020 Mavengere et al. This content is distributed under the terms of the Creative Commons Attribution 4.0 International license.

### Enrichment by sequence type.

In cases where introgression is deleterious, selection will operate most efficiently against regions encoding functional elements (e.g., coding sequences and promoters [49, 80, 81]). To test if a particular type of sequence was more or less prone to appearing in introgressed regions, we partitioned the genome by sequence type into one of the following seven categories using the *P. brasiliensis* Pb18 genome annotations (BioProject accession number PRJNA28733): CDS (coding sequence), 5′ untranslated region [UTR], 3′ UTR, intron, 2-kb upstream inter (intergenic sequence 2 kb upstream of a gene), 10-kb inter (intergenic sequence within 10 kb of a gene excluding 2 kb upstream of a gene), and intergenic (intergenic sequence more than 10 kb from a gene [82]). Introgressions present in more than one individual but with different endpoints among isolates were broken into blocks, and these blocks were treated separately in the permutation test (described immediately below).

We calculated a summary statistic for each of the seven categories using the following definitions: “introgressed percentage” is the percentage of introgressions overlapping a given sequence type that occurred in any of the four possible introgression directions (two different species pairs and two reciprocal directions), “genomic percentage” is the proportion of the genome of any given type of sequence, and “Enrichment” is the ratio of the percentage of a given sequence type that has crossed species boundaries and the percentage of the genome encompassed by the same sequence type. We used a permutation test in which each introgression block was randomly assigned to a new position in the genome to calculate *P* values. For each permutation assay, we calculated the percentage of the randomly reassigned blocks overlapped with type of sequence. We repeated this procedure 10,000 times and generated a null-distribution for enrichment. If introgressions are more likely to occur within a certain type of sequence than in the rest of the genome, enrichment will be greater than 1. Conversely, if introgressions are less likely to occur in a given type of sequence, enrichment will be less than 1. We determined whether introgressions were significantly enriched for any sequence type (i.e., a significant departure from 1), by comparing the observed enrichment and the distribution of resampled enrichments.
