# Supplementary material for: Paracoccidioides Genomes Reflect High Levels of Species Divergence and Little Interspecific Gene Flow
Source: mBio. 2020 Dec 22;11(6):e01999-20. doi: 10.1128/mBio.01999-20 (PMC8534288; doi:10.1128/mBio.01999-20)
Supplement: TABLE S1 [file mbio.01999-20-st001.docx]

**TABLE S1. SRA numbers of the genomes used in this manuscript.**

| **Isolate ID** | **Origin** | **Linage** | **References** | **SRA acession number** |
| --- | --- | --- | --- | --- |
| Pb113 | Amazonas, Brazil | *P. brasiliensis* | (Muñoz JF, Farrer RA *et al.* 2016) | SRR4024728 |
| EPM83 | Colombia | *P. restrepiensis* | (Muñoz JF, Farrer RA *et al.* 2016) | SRR4024727 |
| Pb60855 | Colombia | *P. restrepiensis* | (Muñoz JF, Farrer RA *et al.* 2016) | SRR4024748 |
| PbBac | Colombia | *P. restrepiensis* | (Muñoz JF, Farrer RA *et al.* 2016) | SRR4024736 |
| PbCab | Colombia | *P. restrepiensis* | (Muñoz JF, Farrer RA *et al.* 2016) | SRR4024739 |
| [PbCnha](https://msphere.asm.org/content/1/5/e00213-16#fn-2) | Colombia | *P. restrepiensis* | (Muñoz JF, Farrer RA *et al.* 2016) | SRR4024725 |
| Pb395 | Argentina | *P. restrepiensis* | Current study | SRR9736778 |
| PbJam | Colombia | *P. restrepiensis* | (Muñoz JF, Farrer RA *et al.* 2016) | SRR4024745 |
| [Pb01](https://msphere.asm.org/content/1/5/e00213-16#fn-2) | Goiás, Brazil | *P. lutzii* | (Muñoz JF, Farrer RA *et al.* 2016) | SRR4024789 |
| Pl1578 | Goiás, Brazil | *P. lutzii* | (Muñoz JF, Farrer RA *et al.* 2016) | SRR4023335 |
| ED01 | Goiás, Brazil | *P. lutzii* | (Muñoz JF, Farrer RA *et al.* 2016) | SRR4023518 |
| PlEE | Mato Grosso, Brazil | *P. lutzii* | (Muñoz JF, Farrer RA *et al.* 2016) | SRR4024735 |
| MS1 | Mato Grosso do Sul, Brazil | *P. brasiliensis* | (Muñoz JF, Farrer RA *et al.* 2016) | SRR4024737 |
| MS2 | Mato Grosso do Sul, Brazil | *P. brasiliensis* | (Muñoz JF, Farrer RA *et al.* 2016) | SRR4024738 |
| Pb262 | Minas Gerais, Brazil | *P. americana* | (Muñoz JF, Farrer RA *et al.* 2016) | SRR4024732 |
| PbCaz | Northeast Region, Argentina (NEA) | *P. brasiliensis* | (Muñoz JF, Farrer RA *et al.* 2016) | SRR4024733 |
| Pb1445 | Northeast Region, Argentina (NEA) | *P. brasiliensis* | (Muñoz JF, Farrer RA *et al.* 2016) | SRR4024724 |
| PbBer | Northeast Region, Argentina (NEA) | *P. brasiliensis* | (Muñoz JF, Farrer RA *et al.* 2016) | SRR4024729 |
| [Pb18](https://msphere.asm.org/content/1/5/e00213-16#fn-2) | São Paulo, Brazil | *P. brasiliensis* | (Muñoz JF, Farrer RA *et al.* 2016) | SRR3727854 |
| D03 | São Paulo, Brazil | *P. brasiliensis* | (Muñoz JF, Farrer RA *et al.* 2016) | SRR4024723 |
| D02 | São Paulo, Brazil | *P. brasiliensis* | (Muñoz JF, Farrer RA *et al.* 2016) | SRR4024744 |
| T1F1 | São Paulo, Brazil | *P. brasiliensis* | (Muñoz JF, Farrer RA *et al.* 2016) | SRR4024731 |
| T15N1 | São Paulo, Brazil | *P. brasiliensis* | (Muñoz JF, Farrer RA *et al.* 2016) | SRR4024741 |
| T16B1 | São Paulo, Brazil | *P. brasiliensis* | (Muñoz JF, Farrer RA *et al.* 2016) | SRR4024730 |
| Pb339 | São Paulo, Brazil | *P. restrepiensis* | (Muñoz JF, Farrer RA *et al.* 2016) | SRR4024750 |
| [Pb03](https://msphere.asm.org/content/1/5/e00213-16#fn-2) | São Paulo, Brazil | *P. americana* | (Muñoz JF, Farrer RA *et al.* 2016) | SRR4024790 |
| T10B1 | São Paulo, Brazil | *P. americana* | (Muñoz JF, Farrer RA *et al.* 2016) | SRR4024743 |
| Pb304 | Venezuela | *P. venezuelensis* | (Teixeira *et al.* 2020) | SRR9736751 |
| Pb305 | Venezuela | *P. venezuelensis* | (Teixeira *et al.* 2020) | SRR9736752 |
| Pb307 | Venezuela | *P. venezuelensis* | (Teixeira *et al.* 2020) | SRR9736753 |
| Pb309 | Venezuela | *P. venezuelensis* | (Teixeira *et al.* 2020) | SRR9736754 |
| Pb384 | Venezuela | *P. venezuelensis* | (Teixeira *et al.* 2020) | SRR9736749 |
| Pb387 | Venezuela | *P. venezuelensis* | (Teixeira *et al.* 2020) | SRR9736750 |
| Pb444 | Venezuela | *P. venezuelensis* | (Teixeira *et al.* 2020) | SRR9736748 |
| [Pb300](https://msphere.asm.org/content/1/5/e00213-16#fn-2) | Venezuela | *P. venezuelensis* | (Muñoz JF, Farrer RA *et al.* 2016) | SRR4024740 |
| Pb02 | Venezuela | *P. americana* | (Muñoz JF, Farrer RA *et al.* 2016) | SRR4023673 |
| Pb66 | Brazil | *P. brasiliensis* | (Muñoz JF, Farrer RA *et al.* 2016) | SAMN05171529 |
