# Supplementary figures and images for: Paracoccidioides Genomes Reflect High Levels of Species Divergence and Little Interspecific Gene Flow
Source: mBio. 2020 Dec 22;11(6):e01999-20. doi: 10.1128/mBio.01999-20 (PMC8534288; doi:10.1128/mBio.01999-20)

**Supercontig\_1.2 2512546 – 2565821;  
53.3kb 11 inds;  
max\_introgres\_snps = 63**

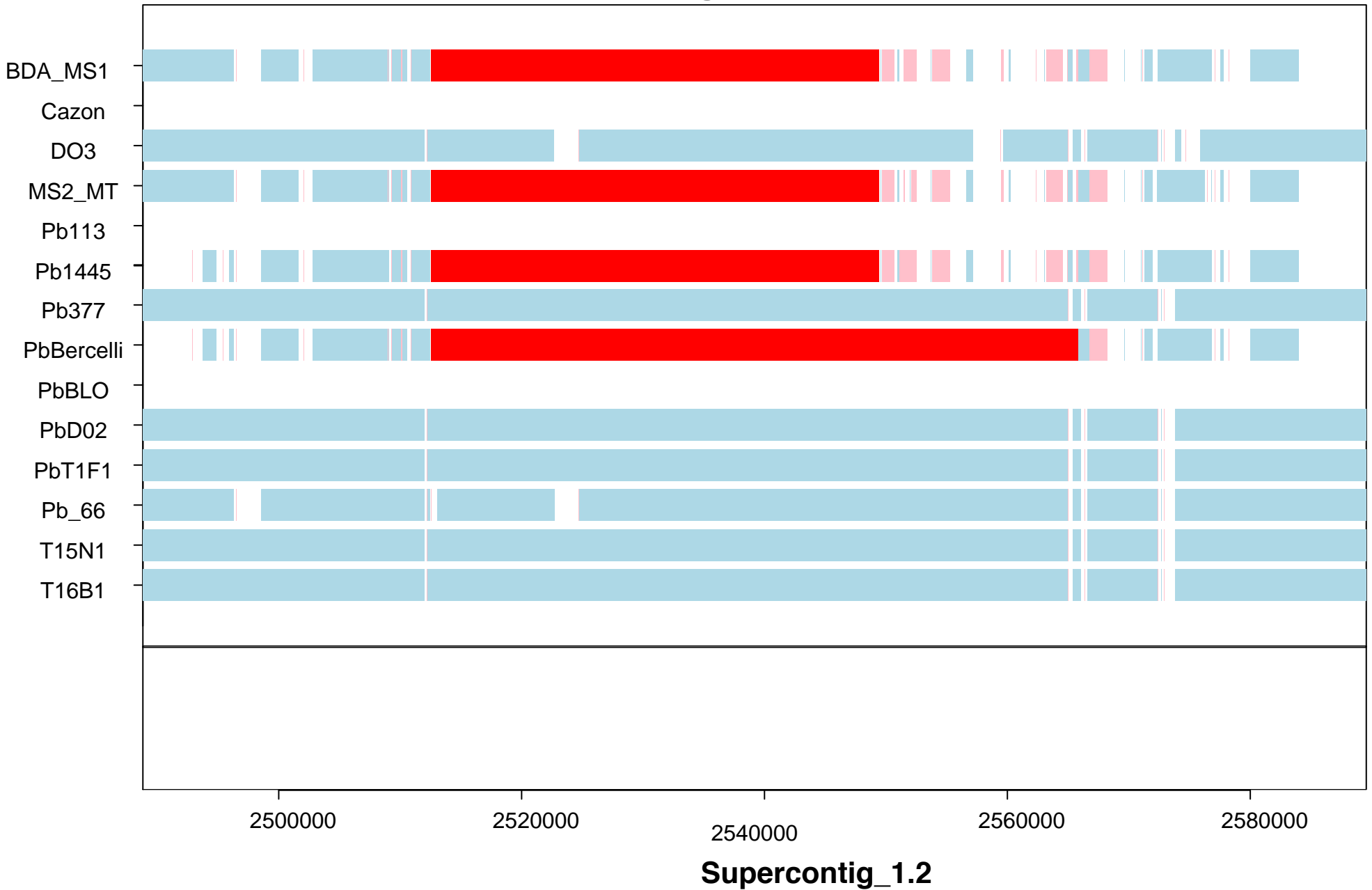

Supplement: FIG S1 [file mbio.01999-20-sf001.pdf]

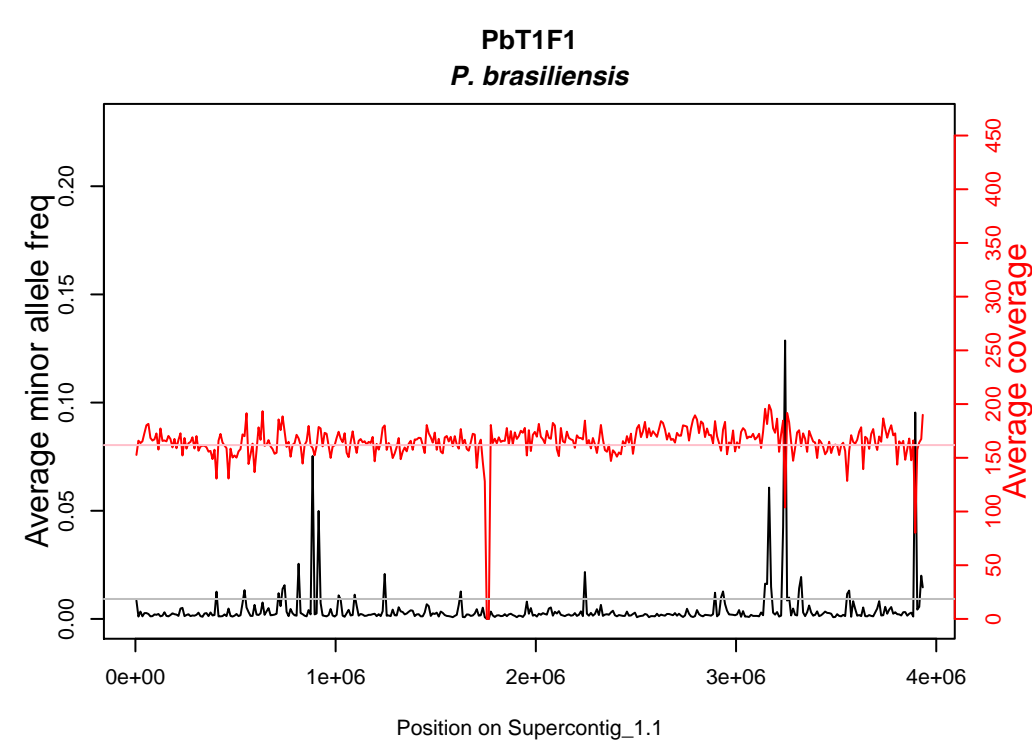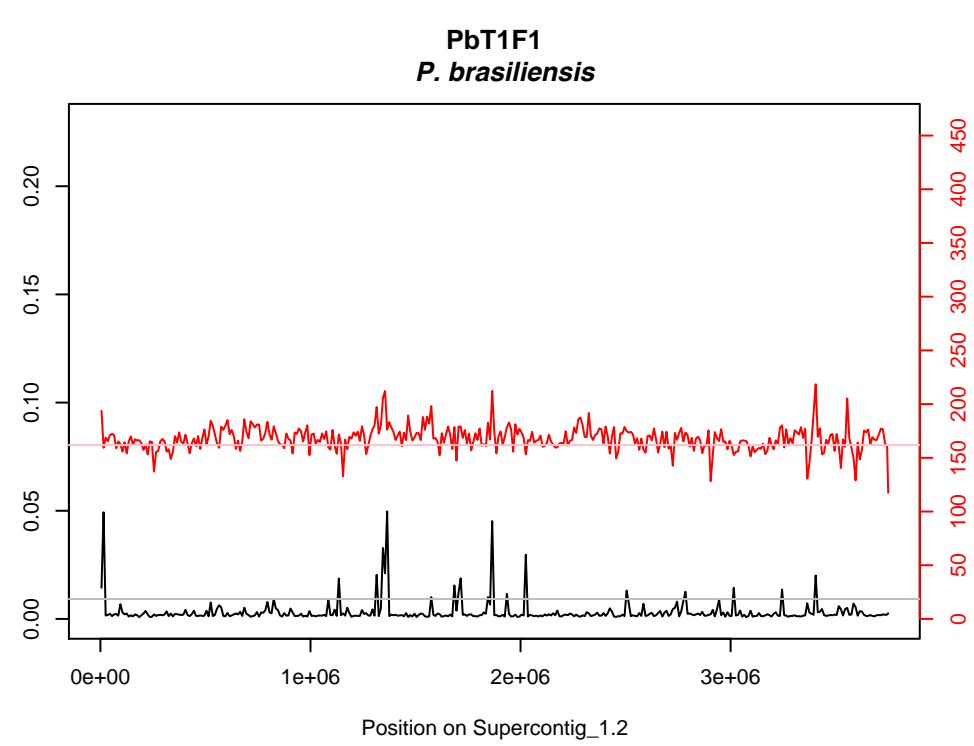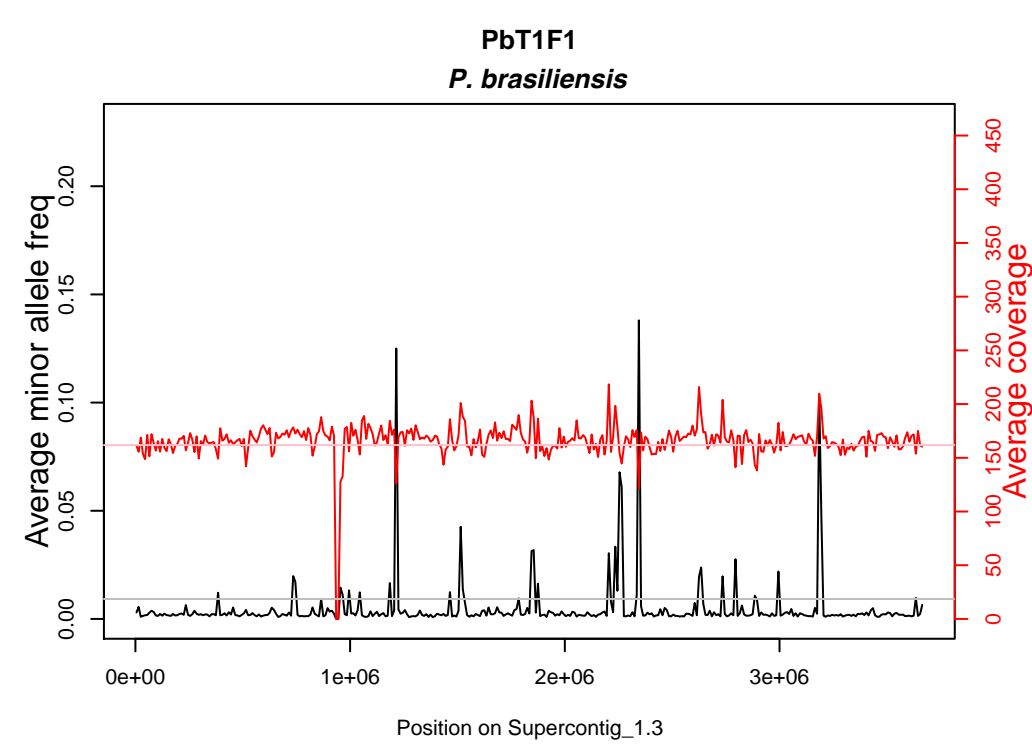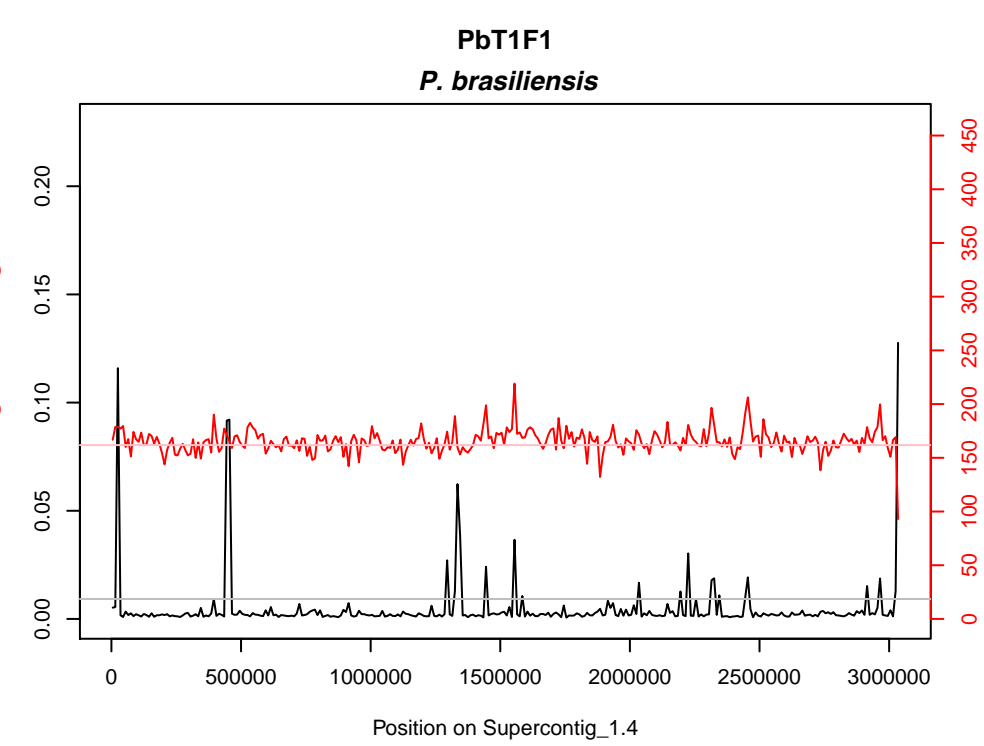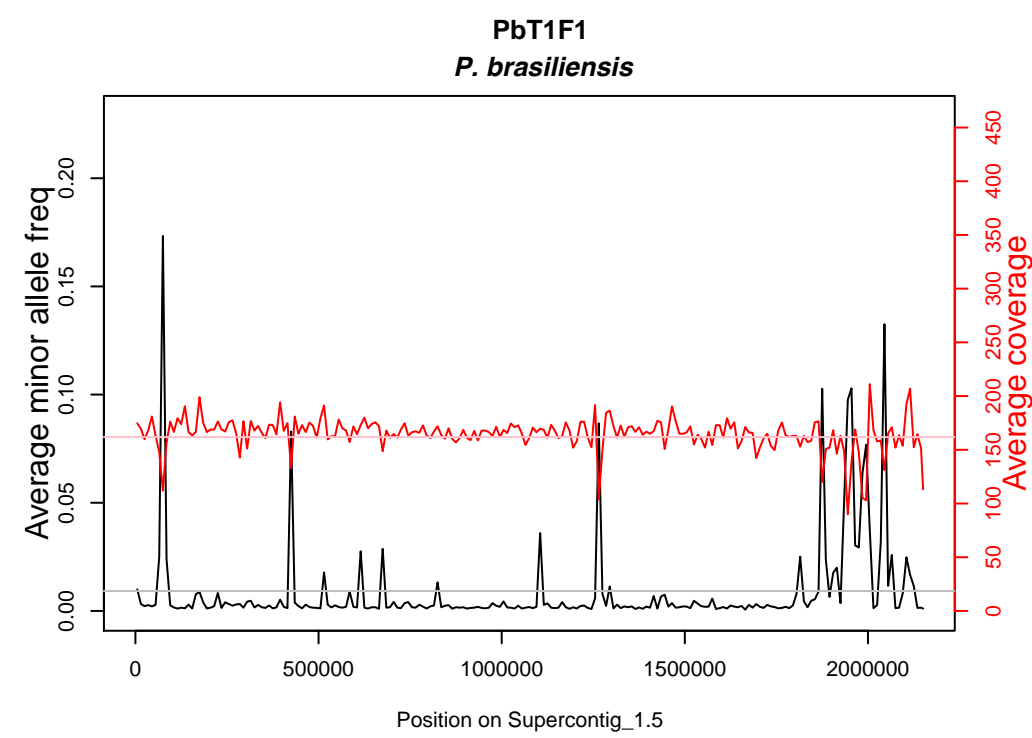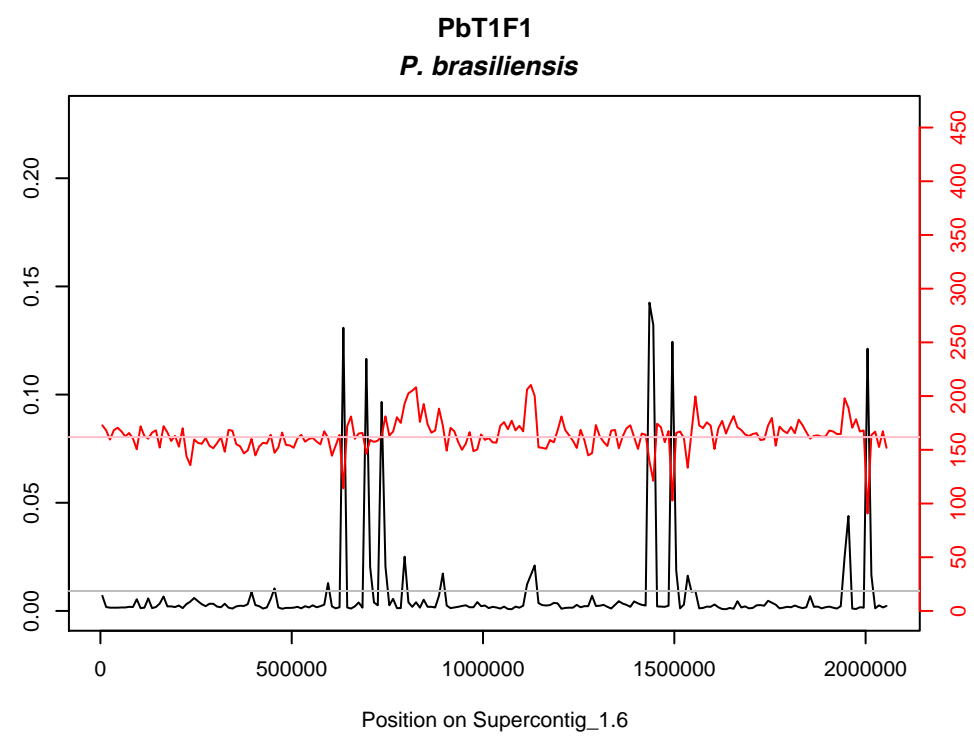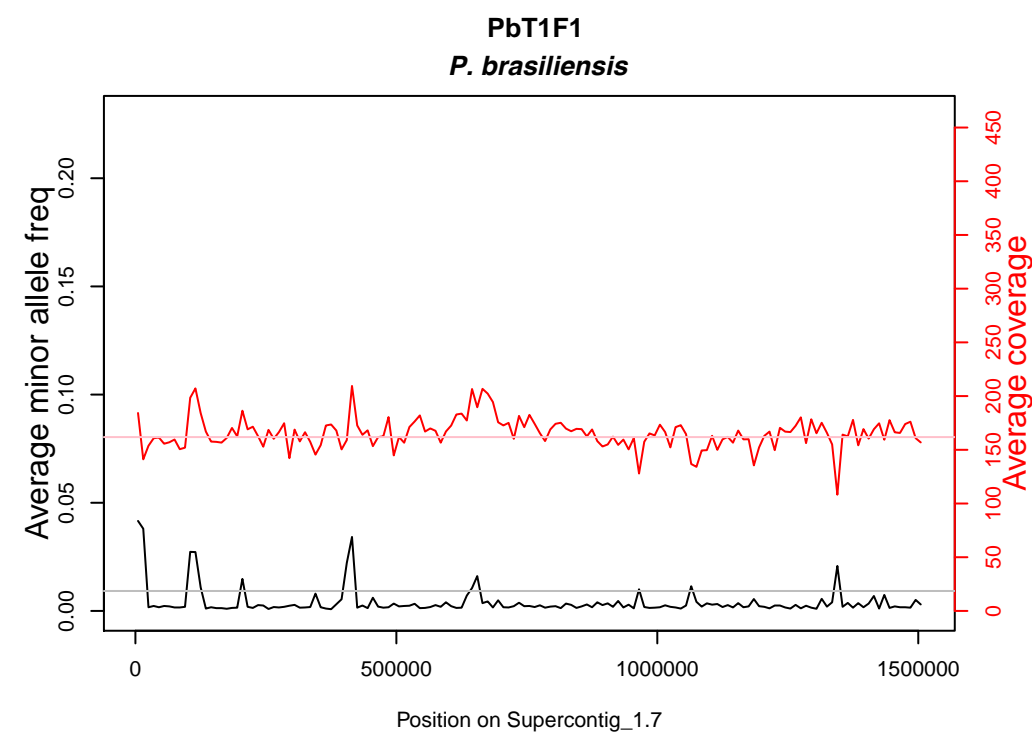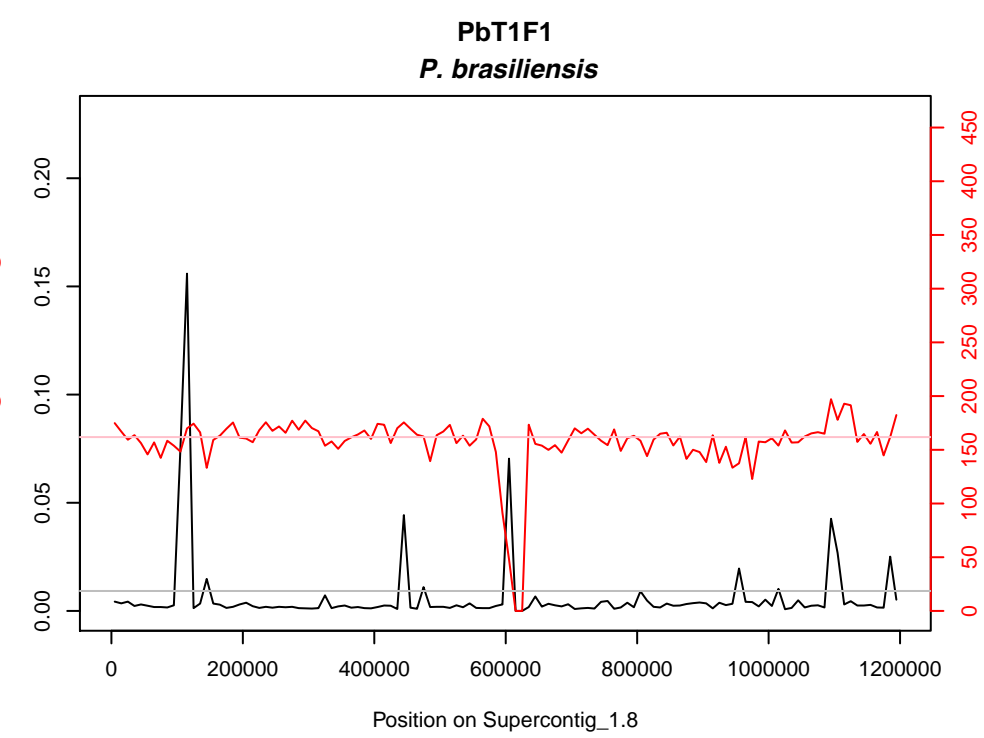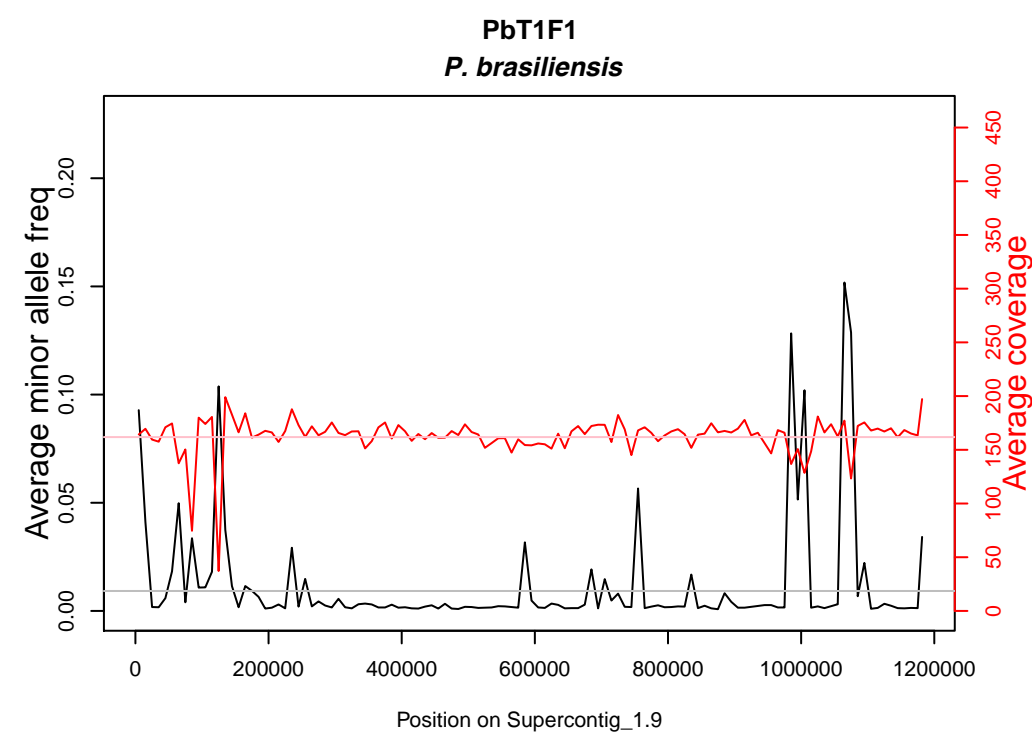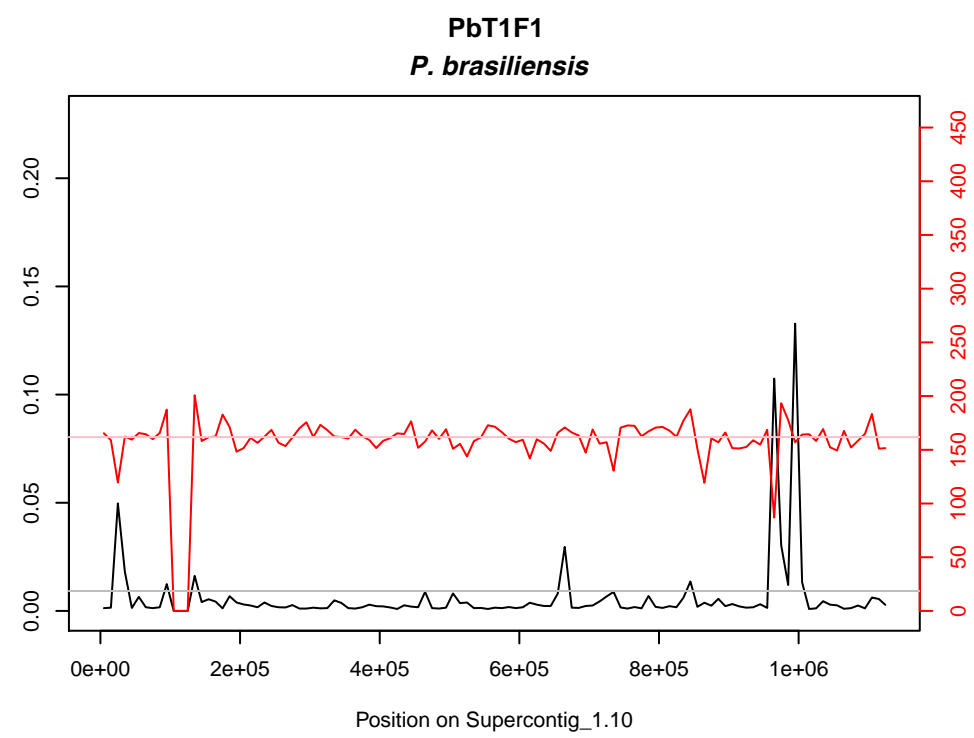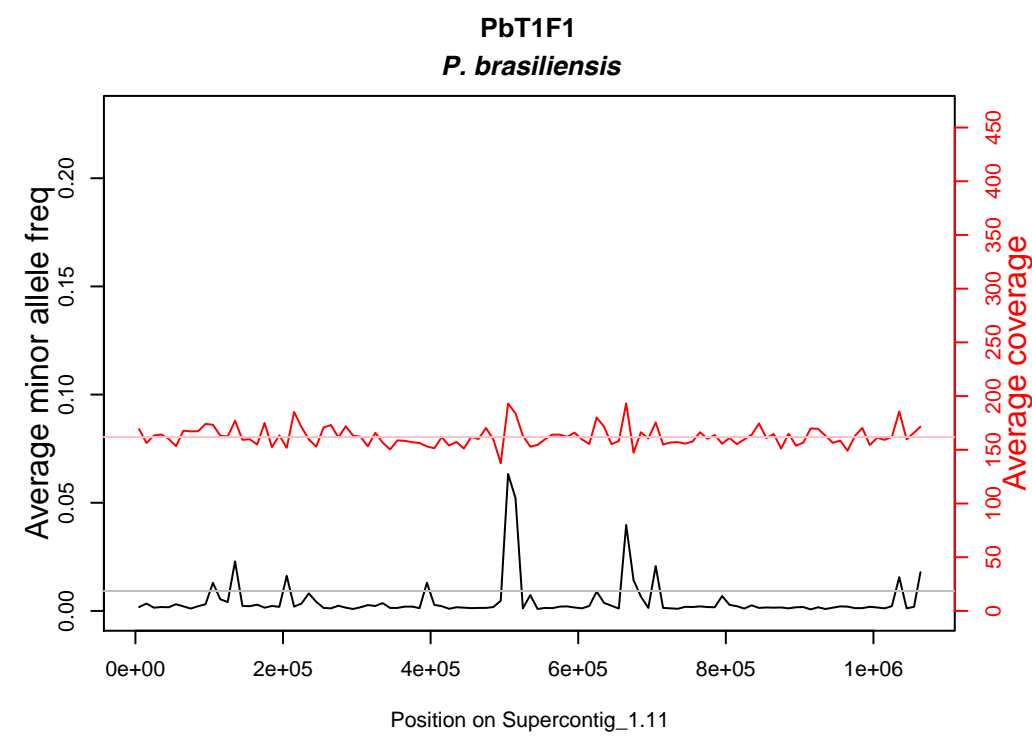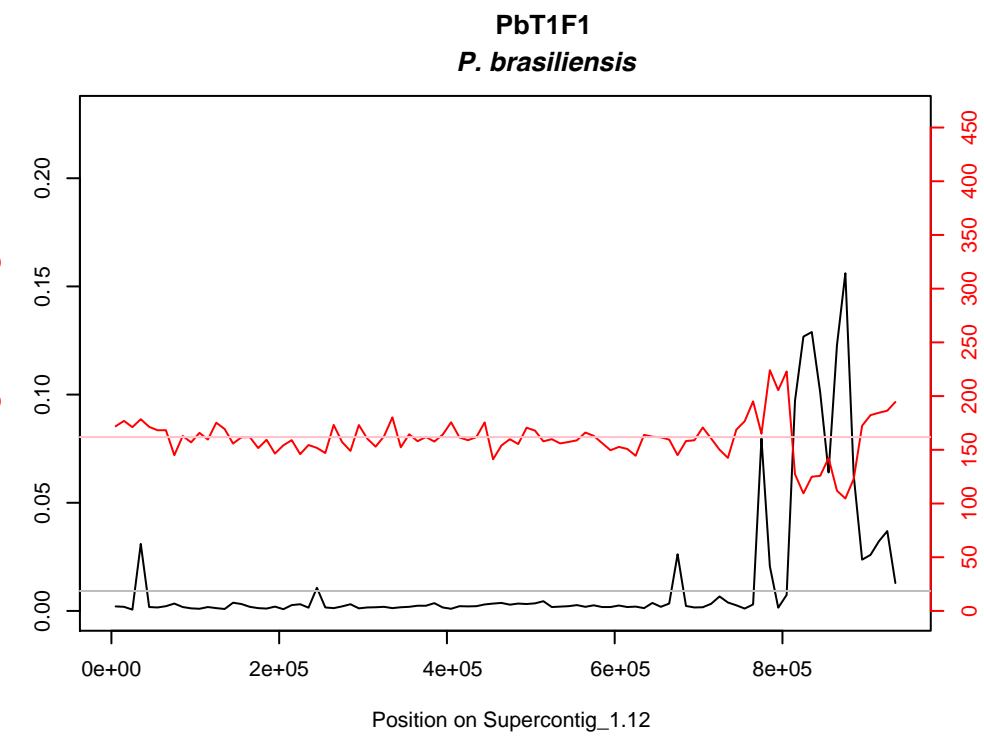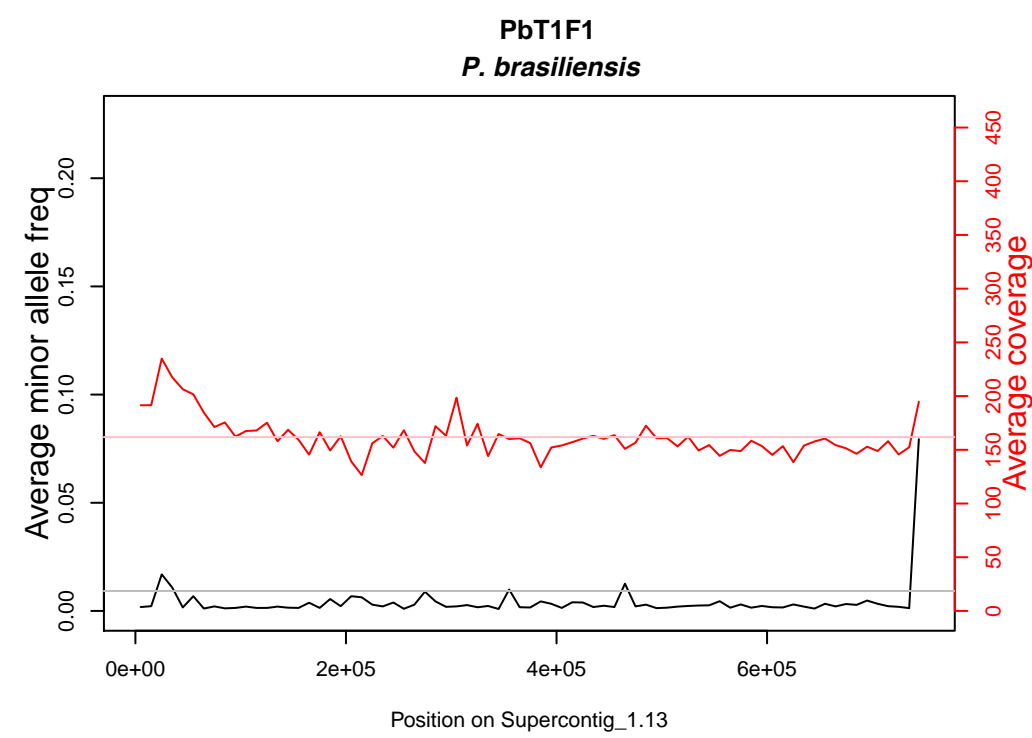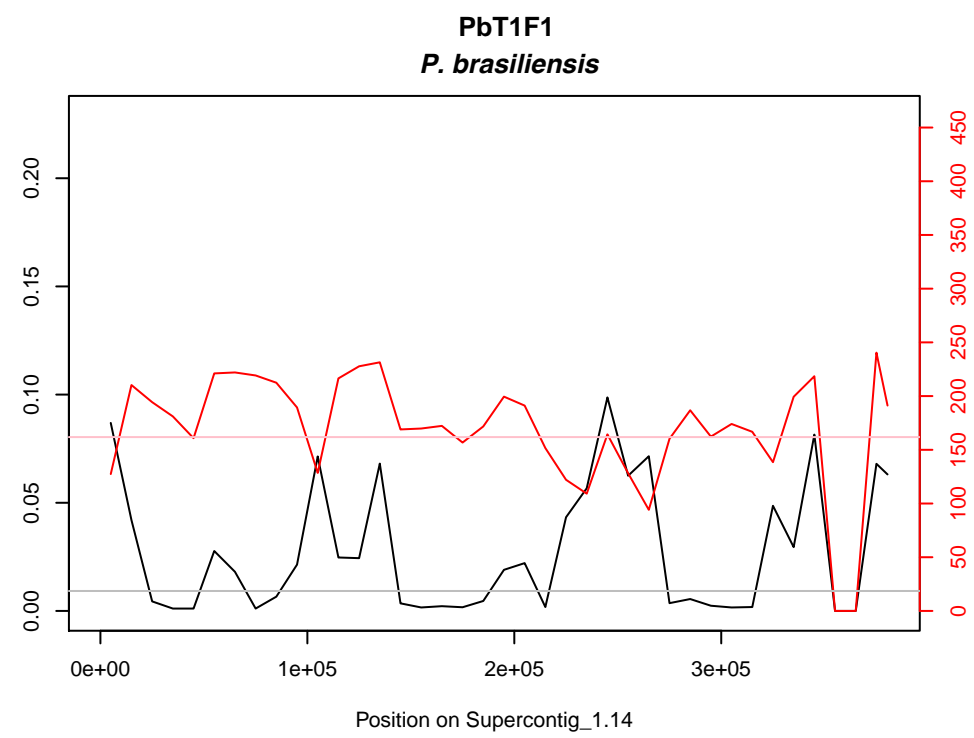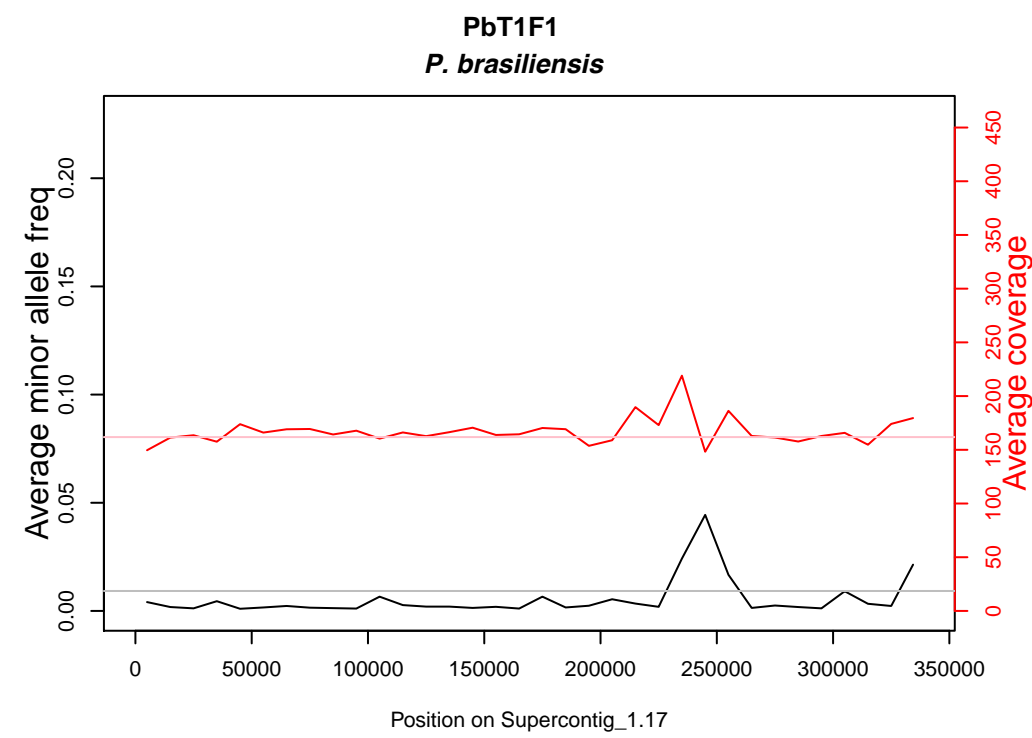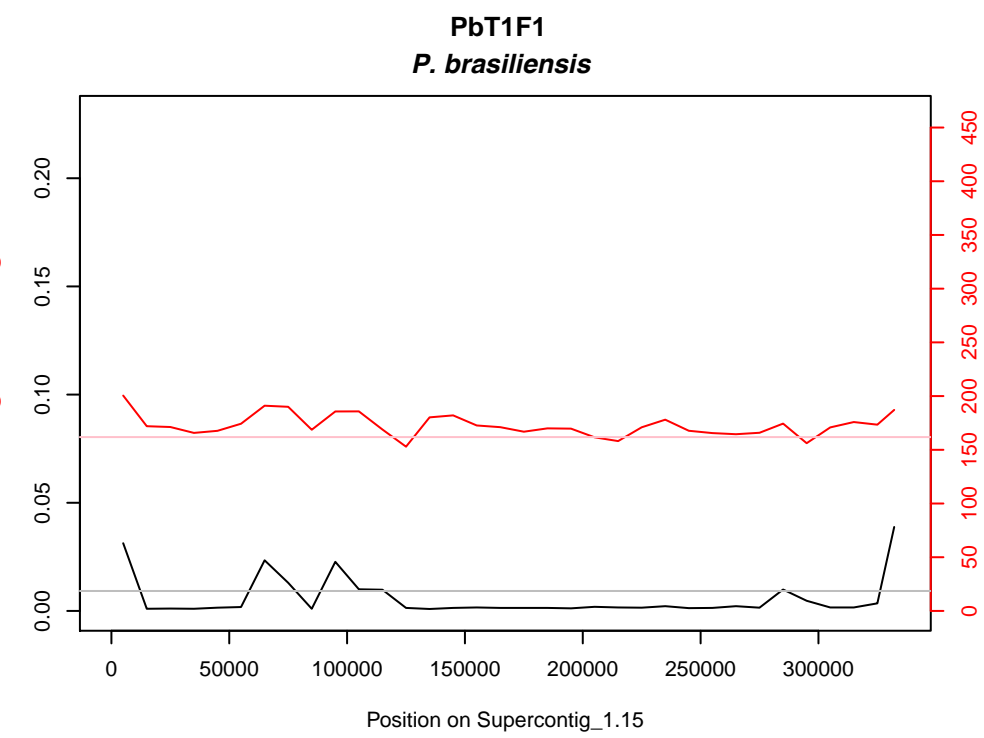

Supplement: FIG S3 [file mbio.01999-20-sf003.pdf]

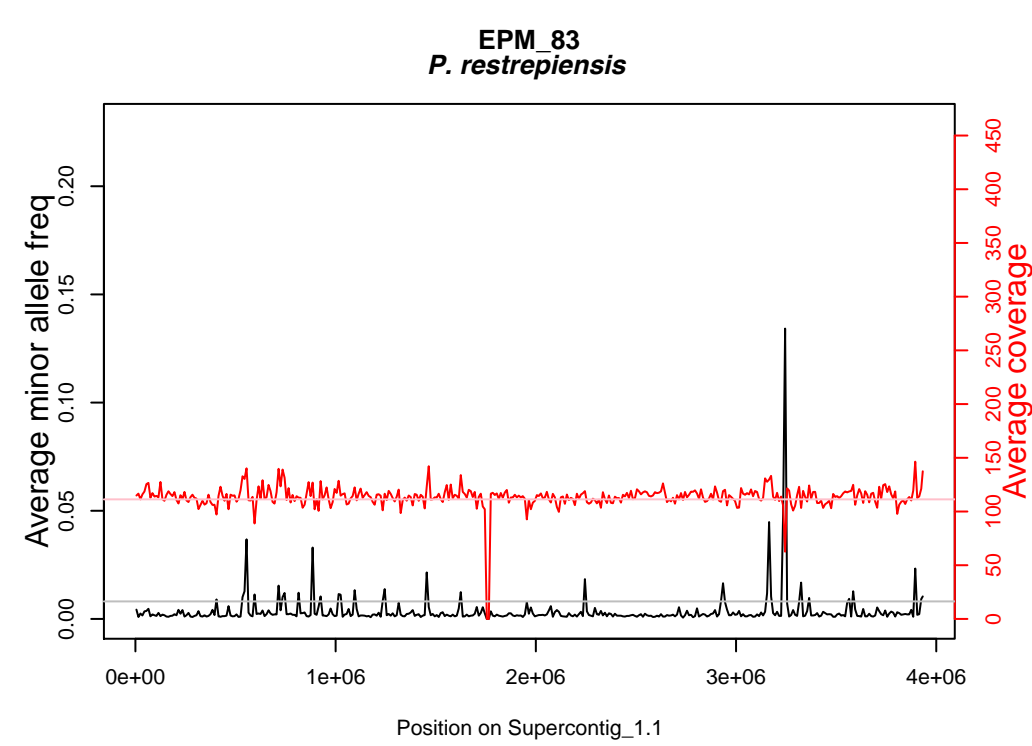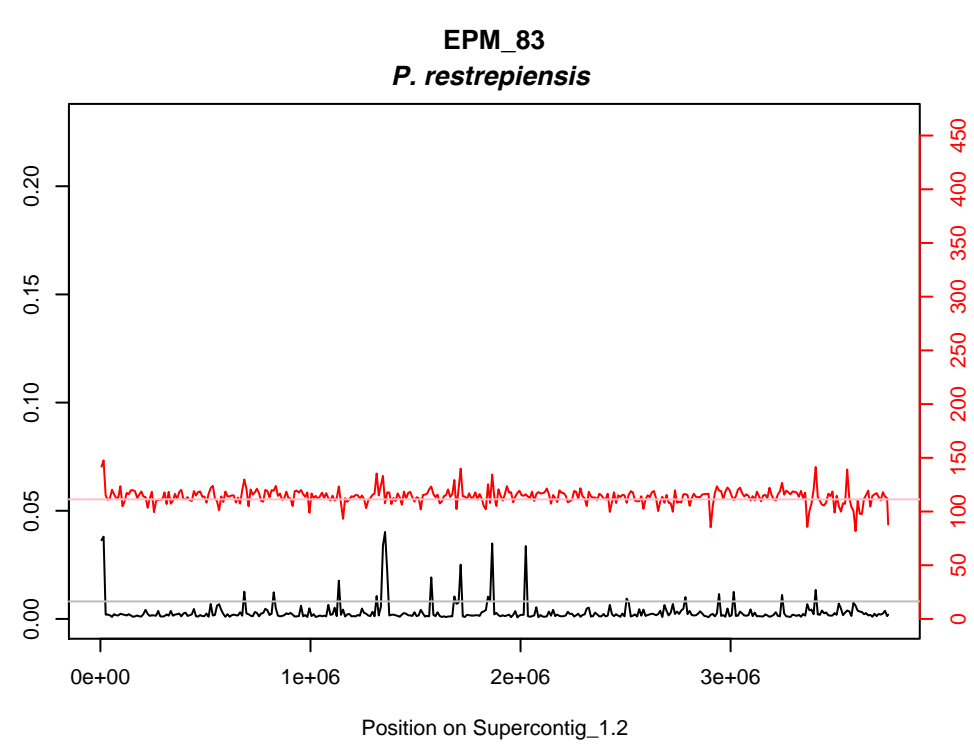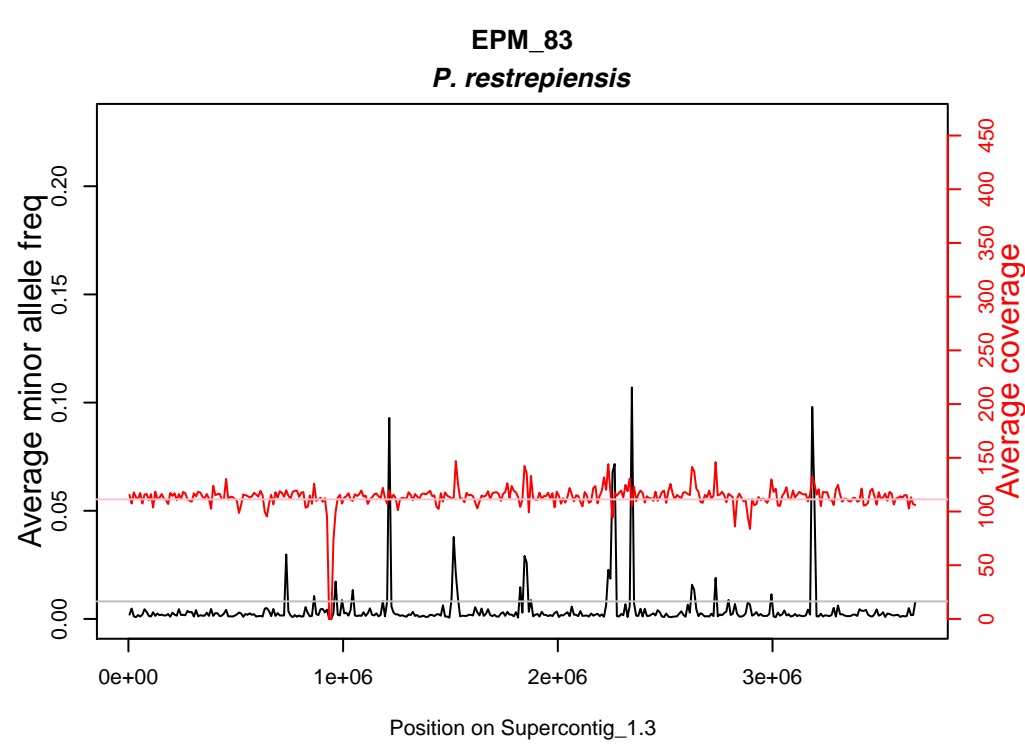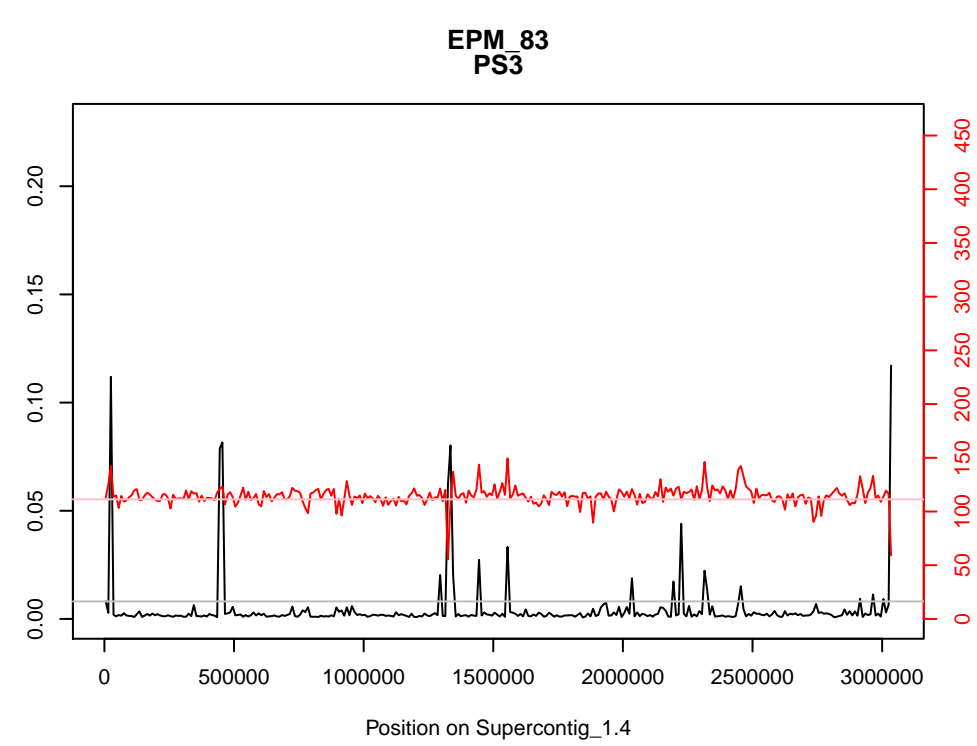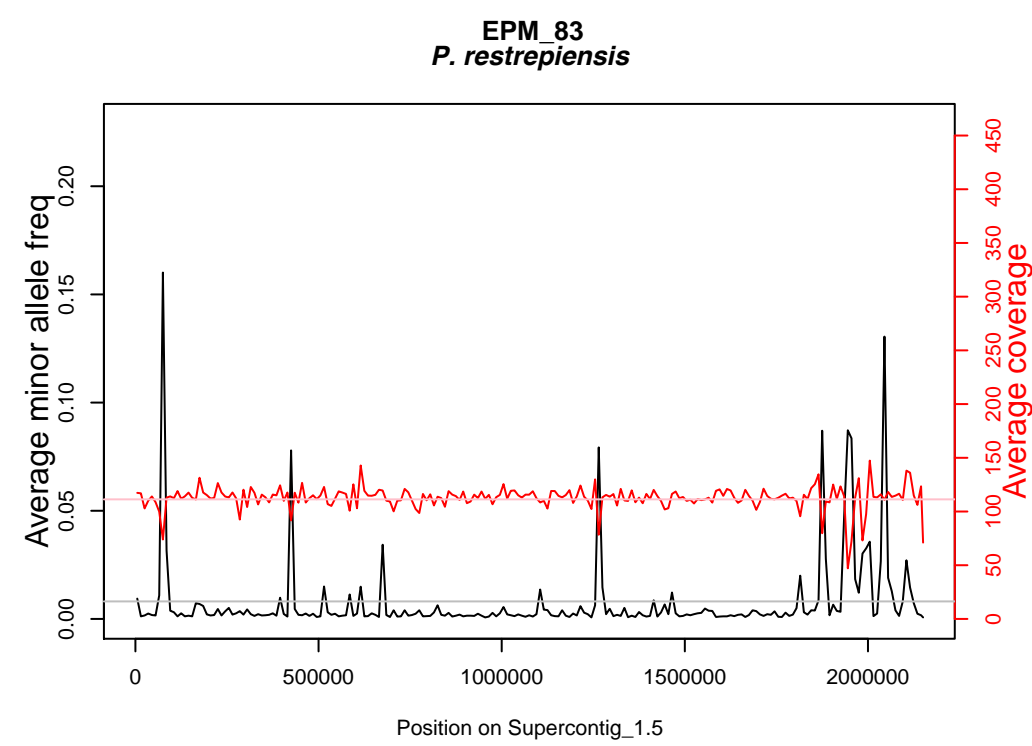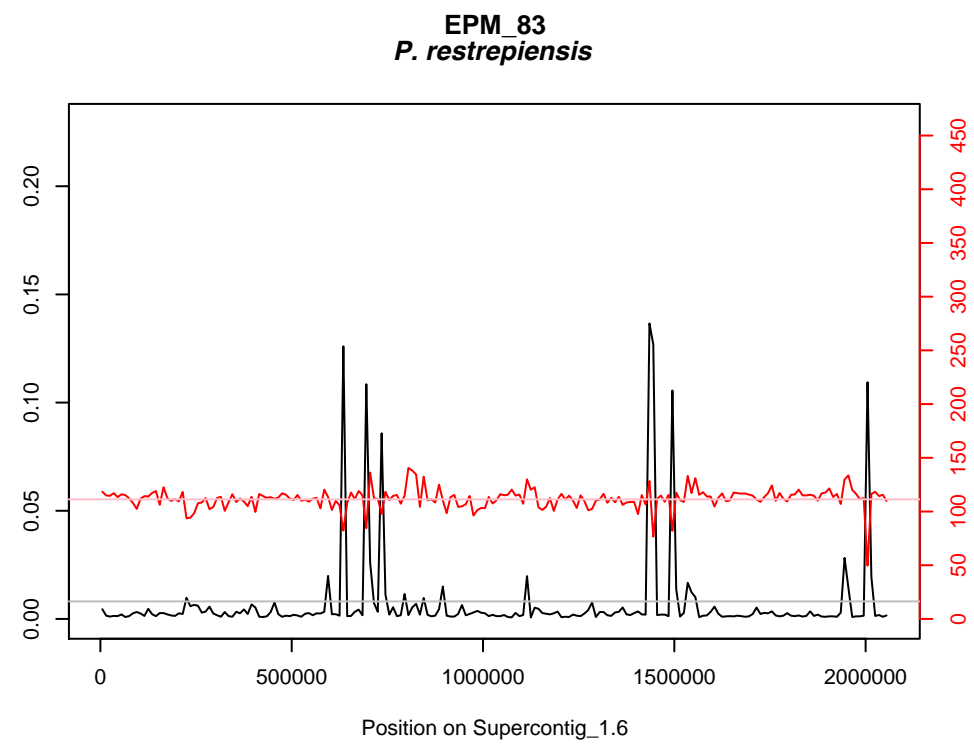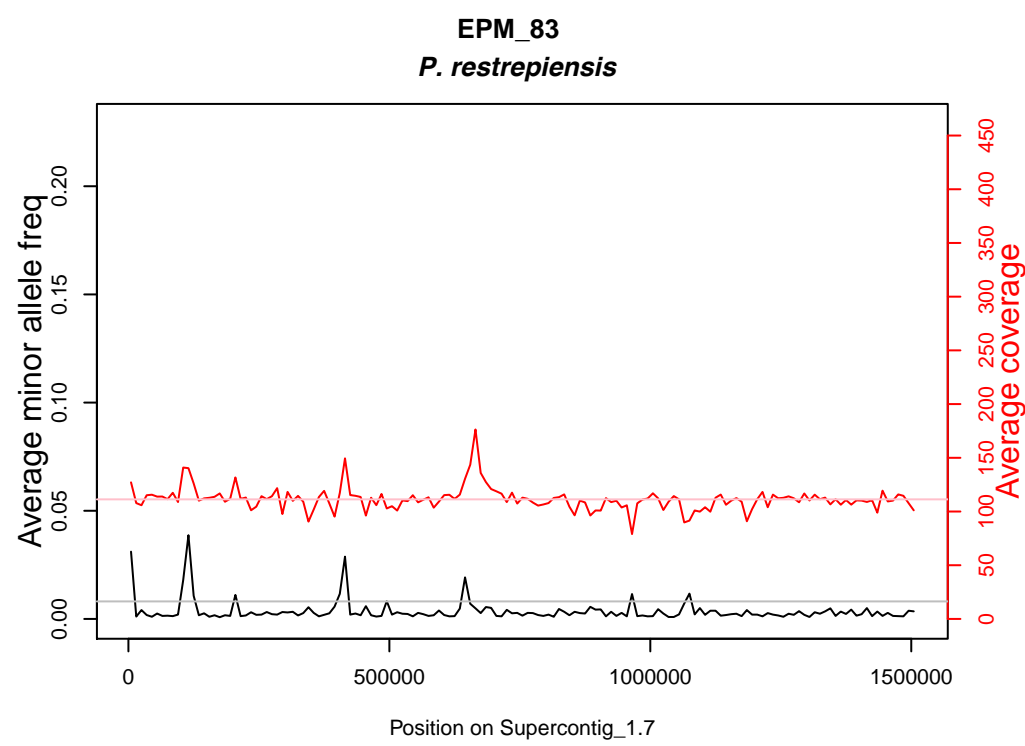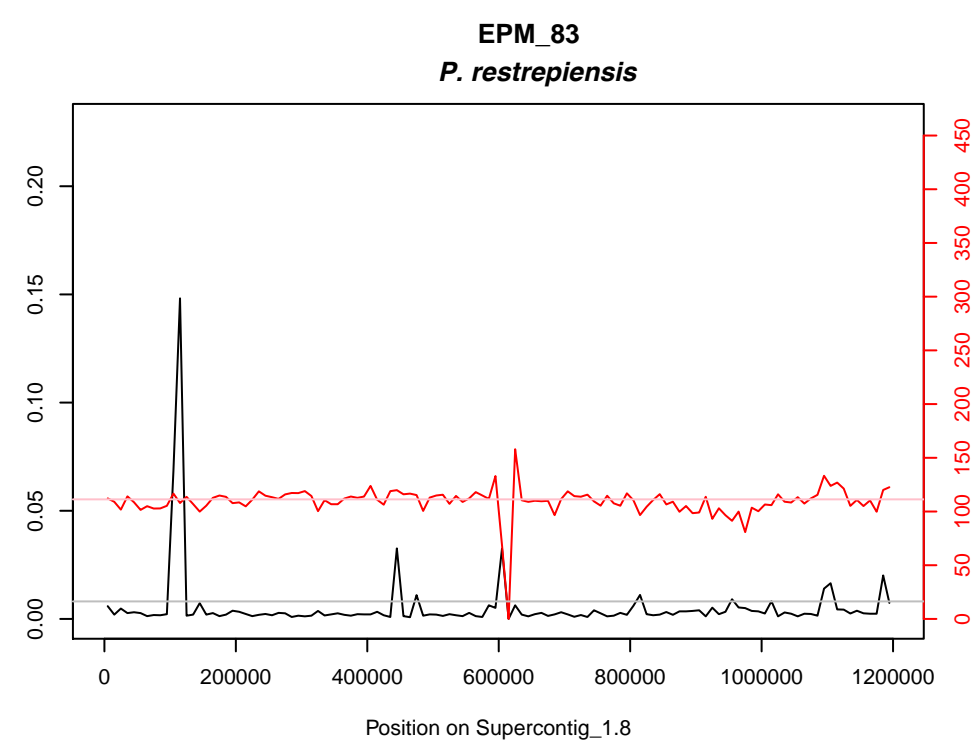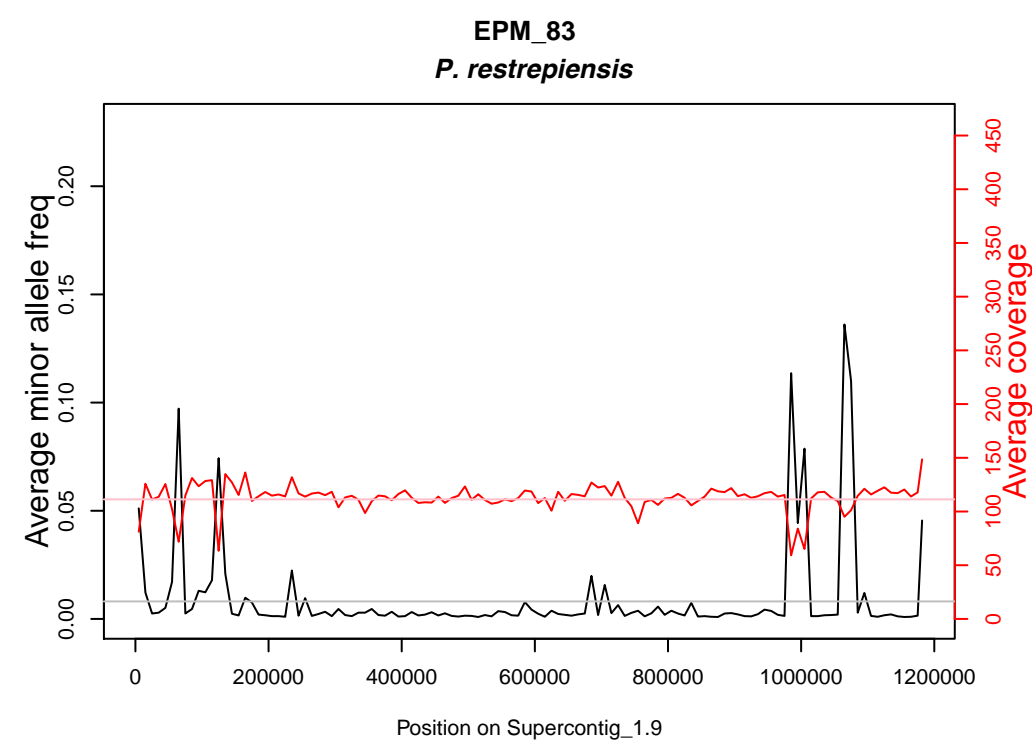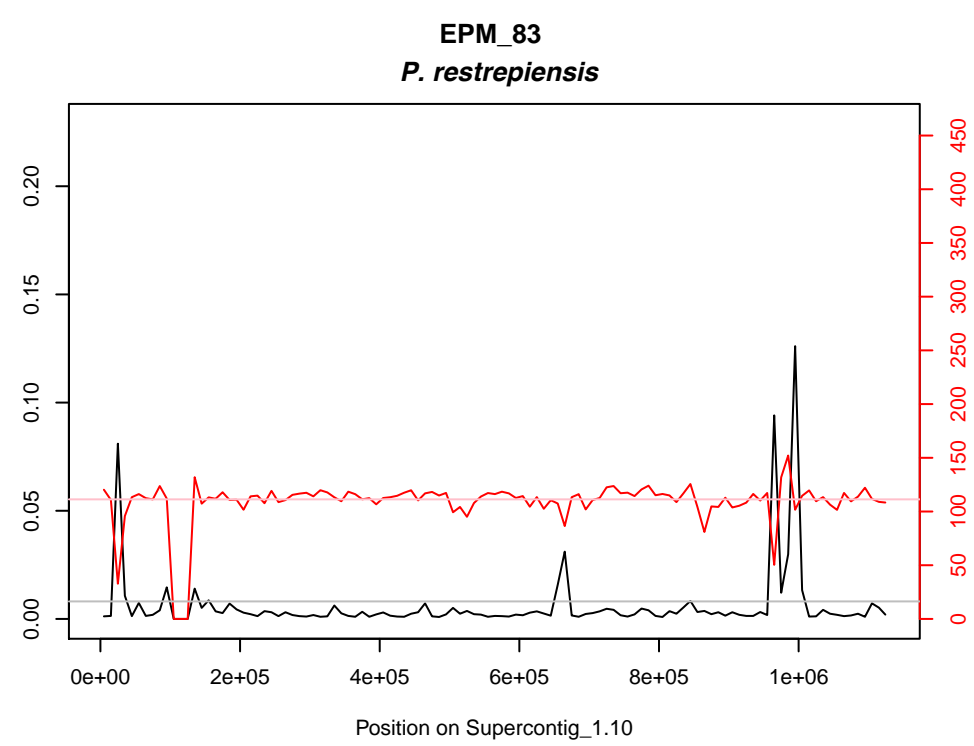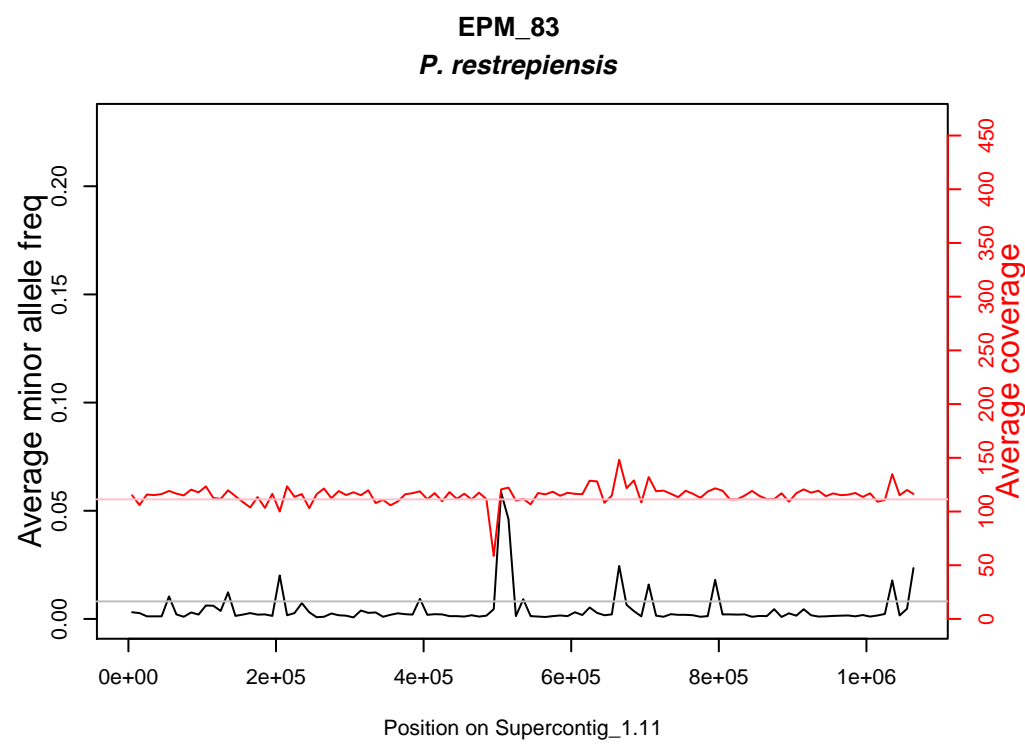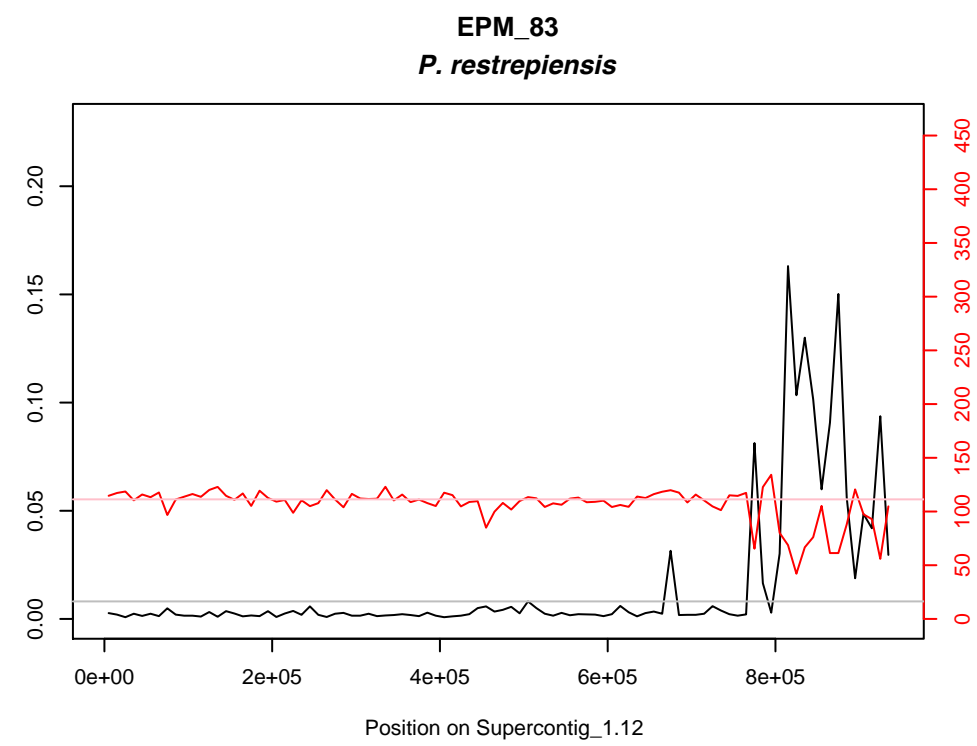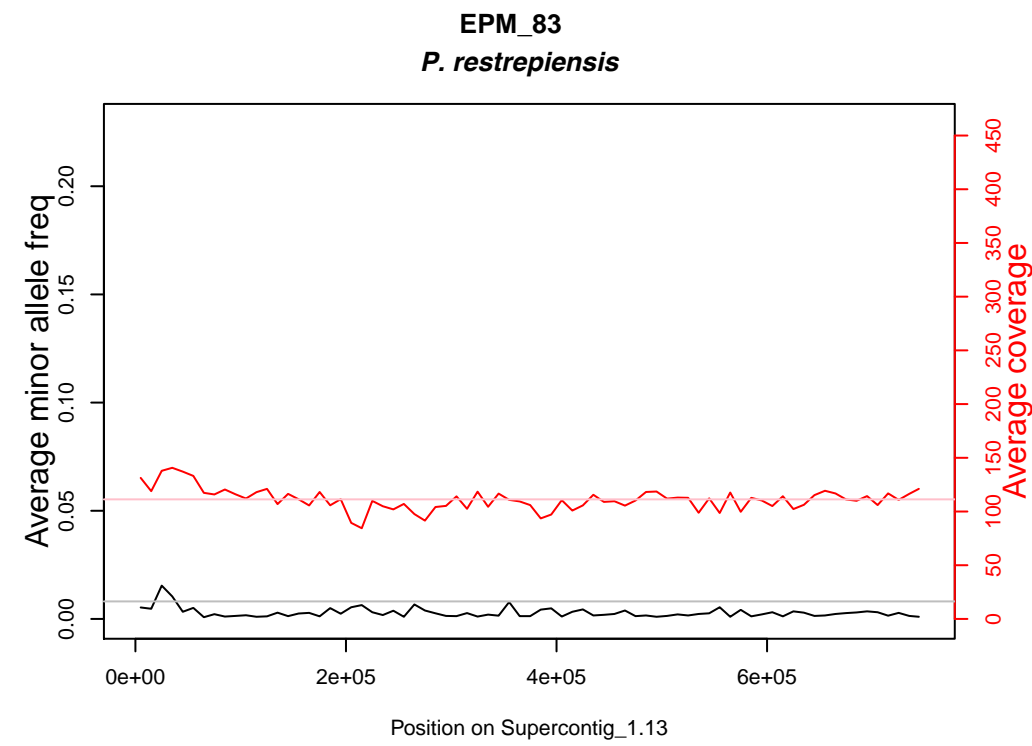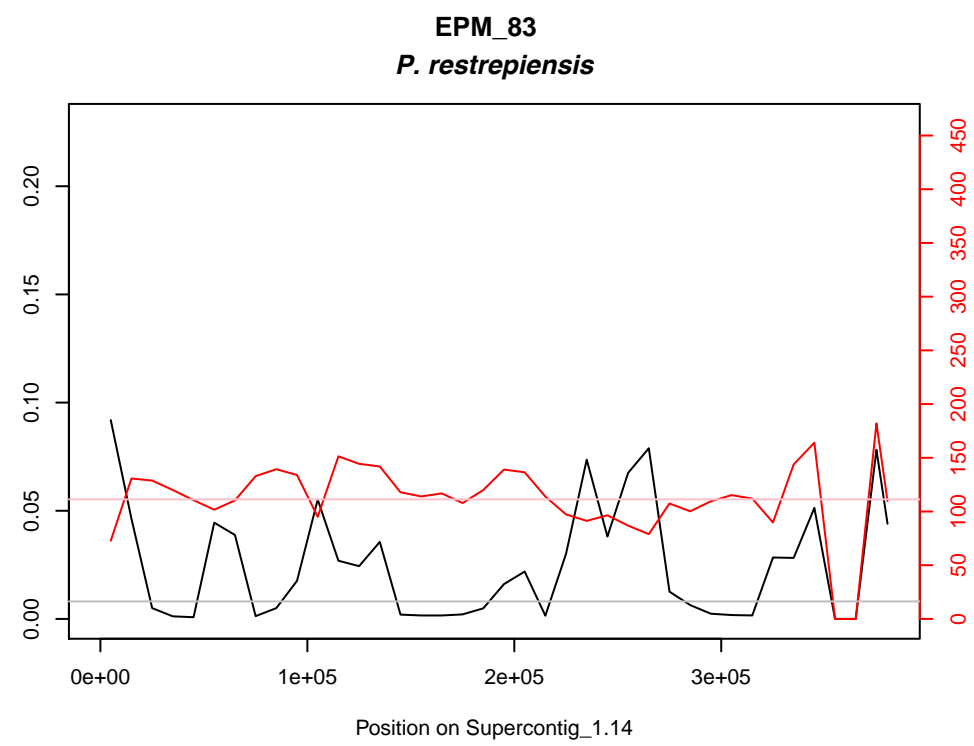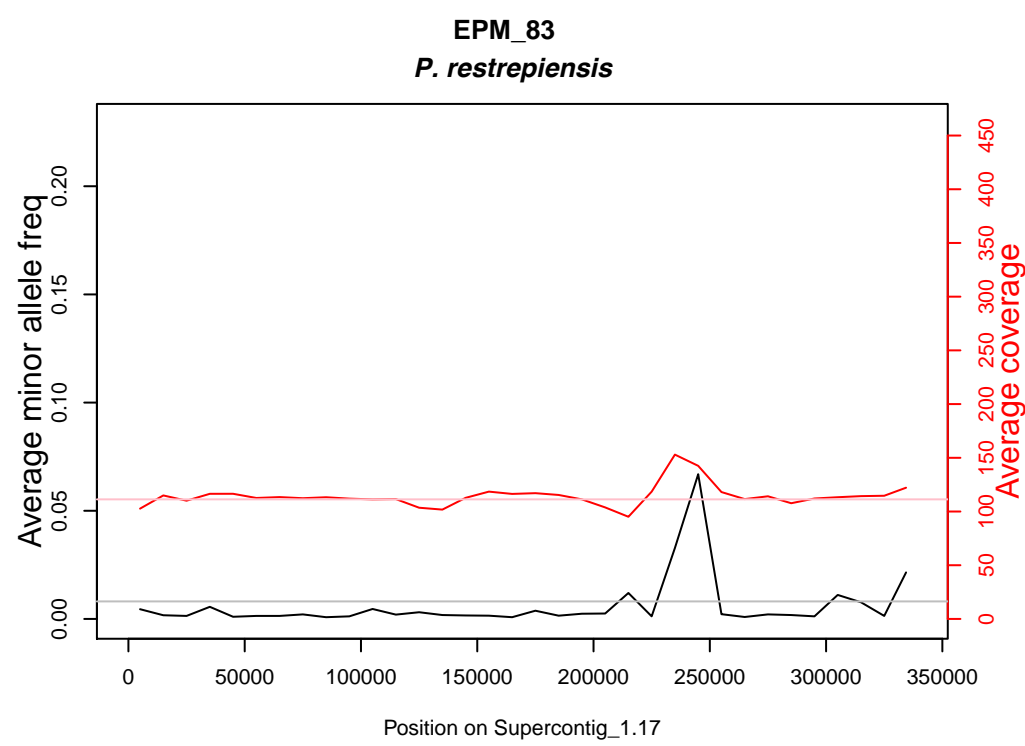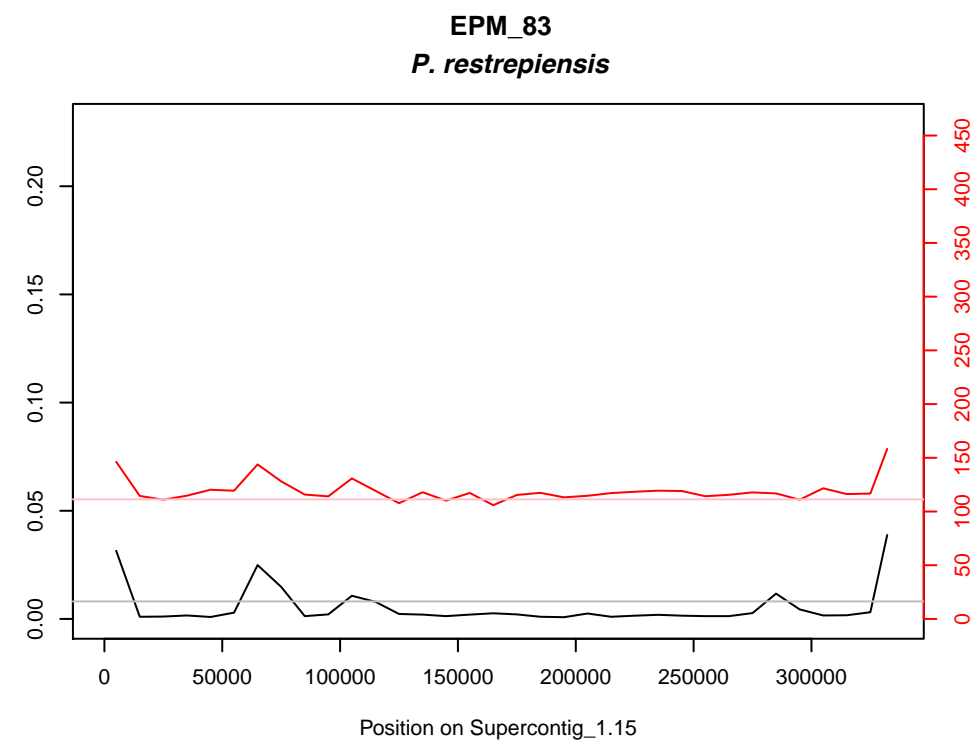

Supplement: FIG S4 [file mbio.01999-20-sf004.pdf]

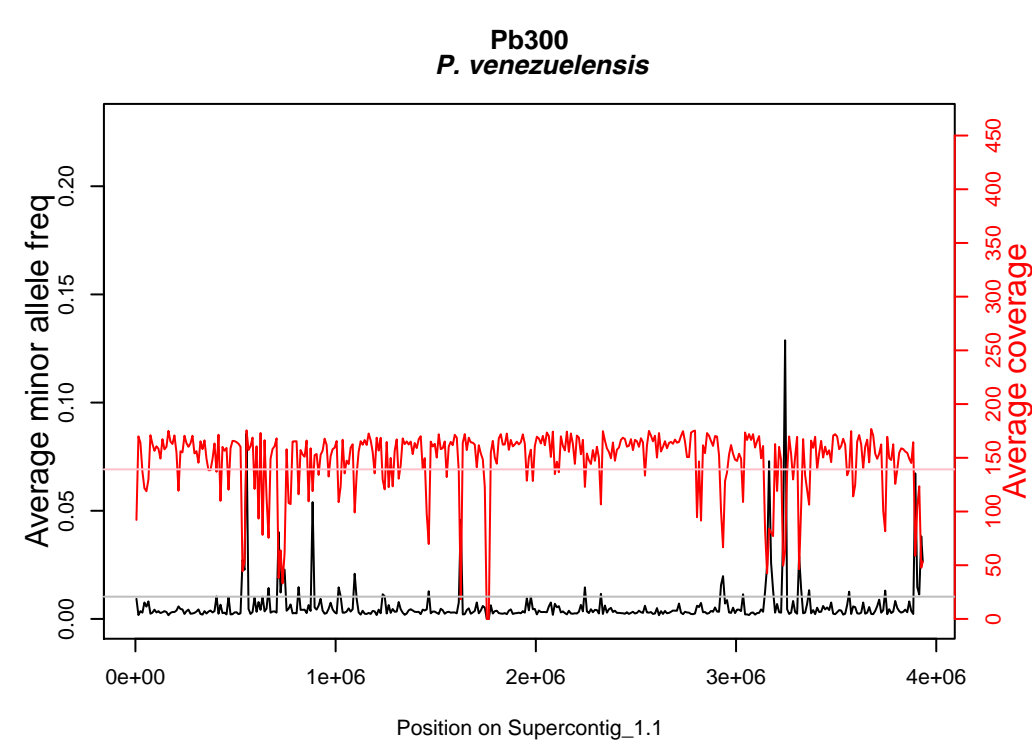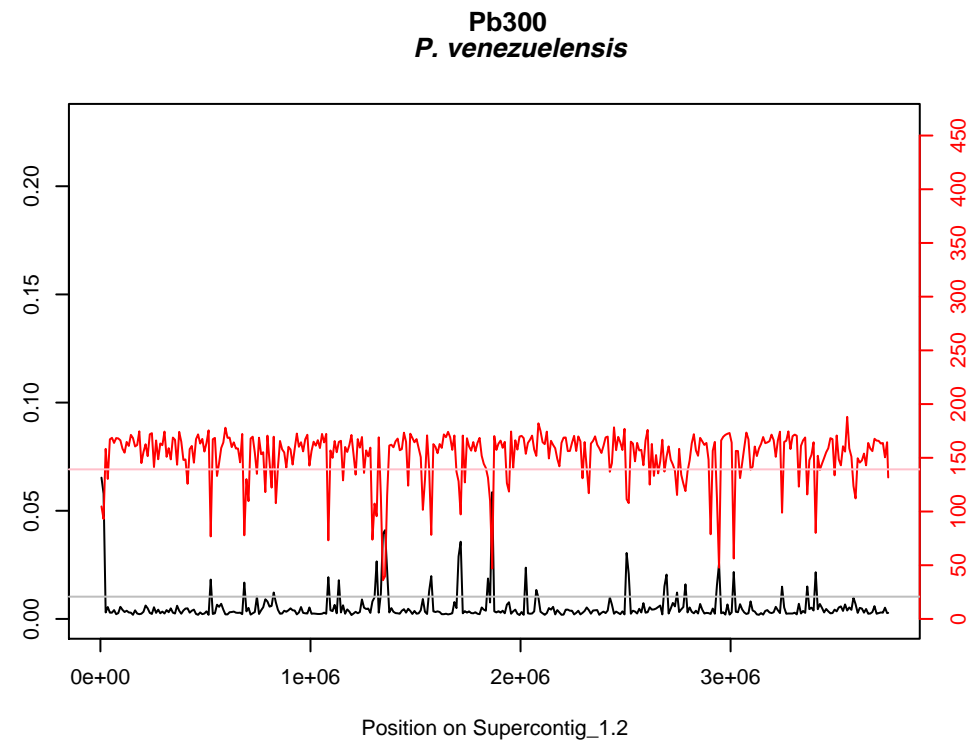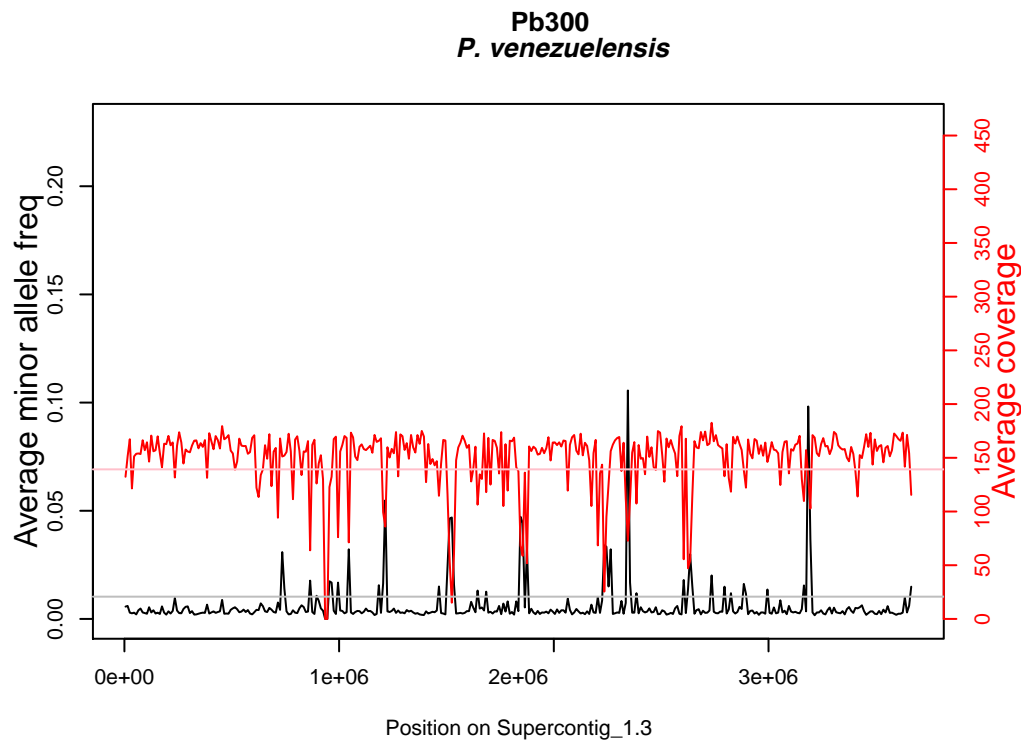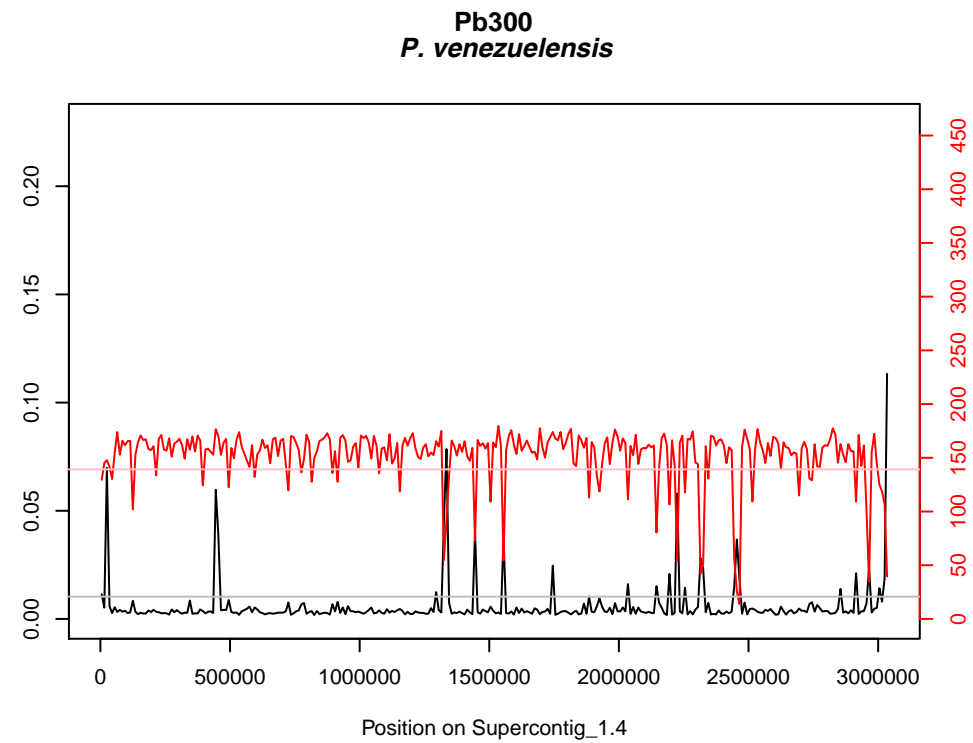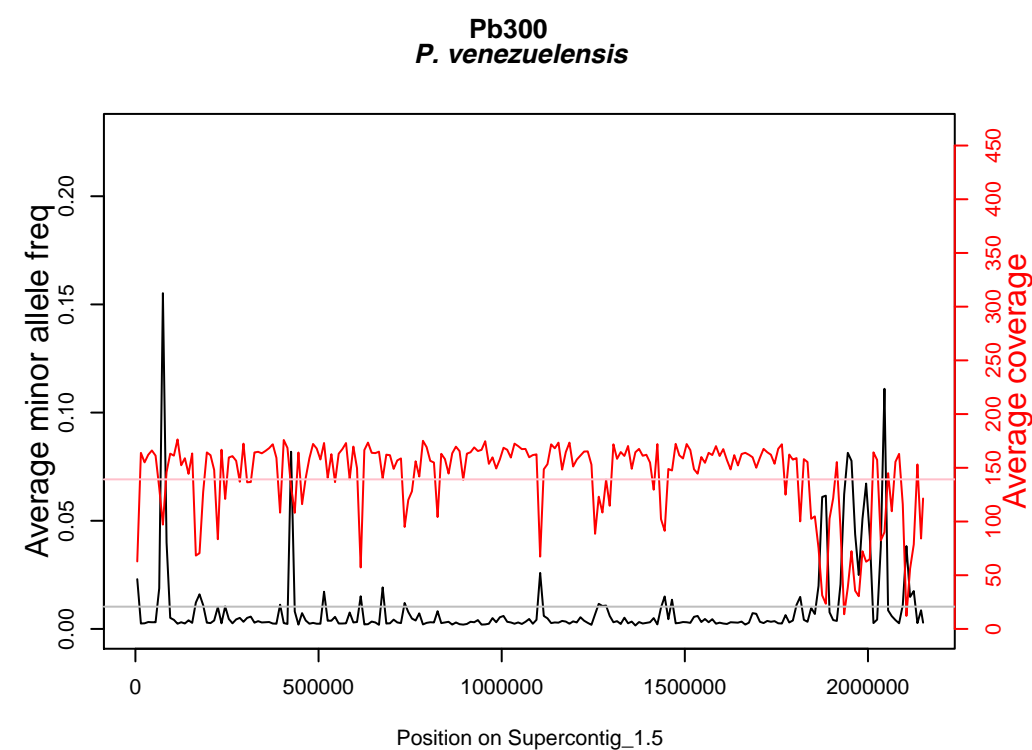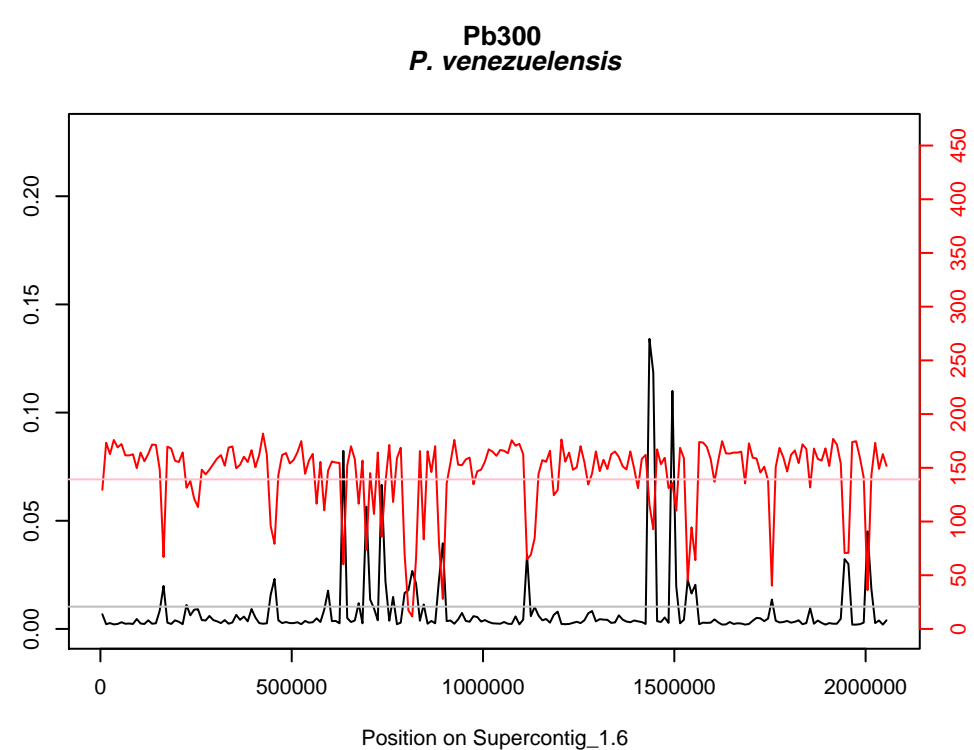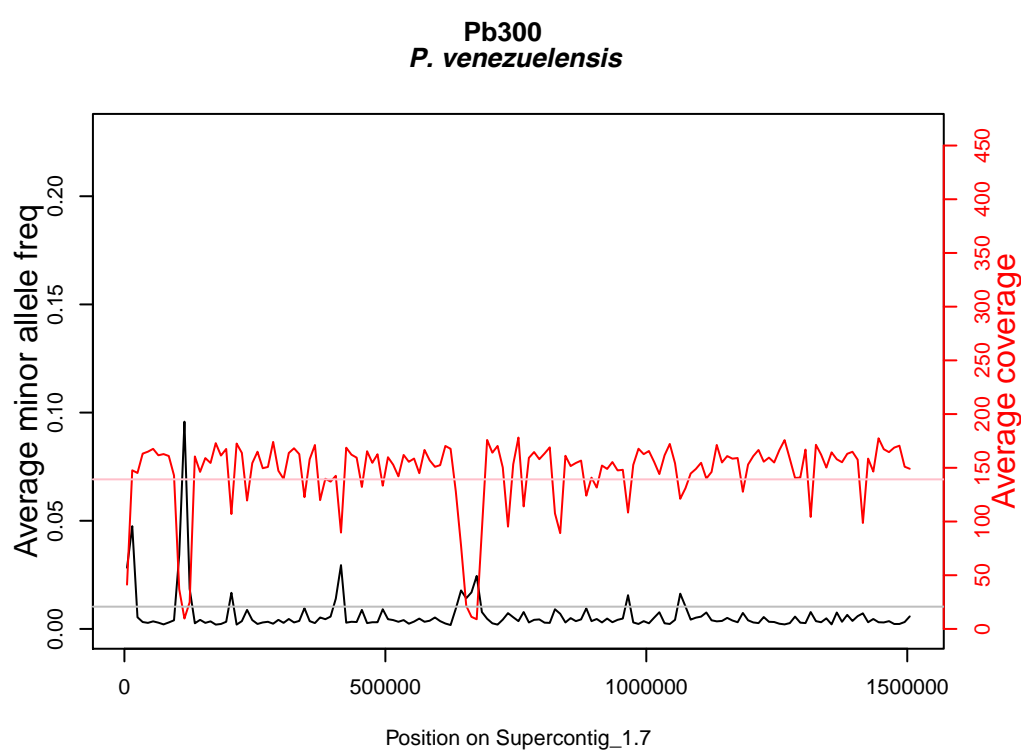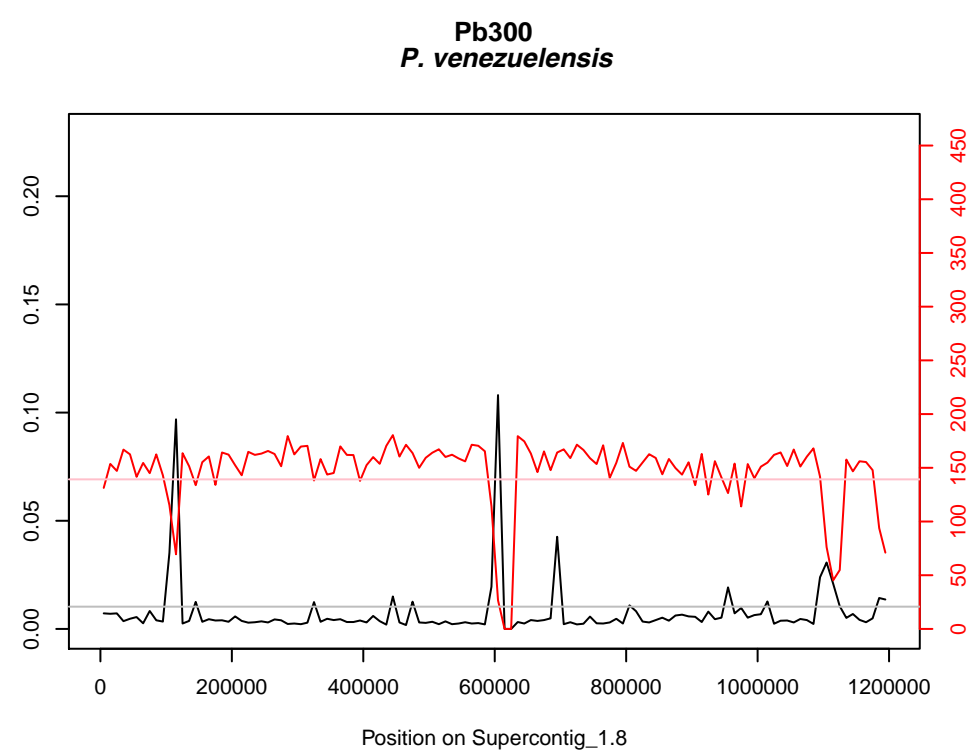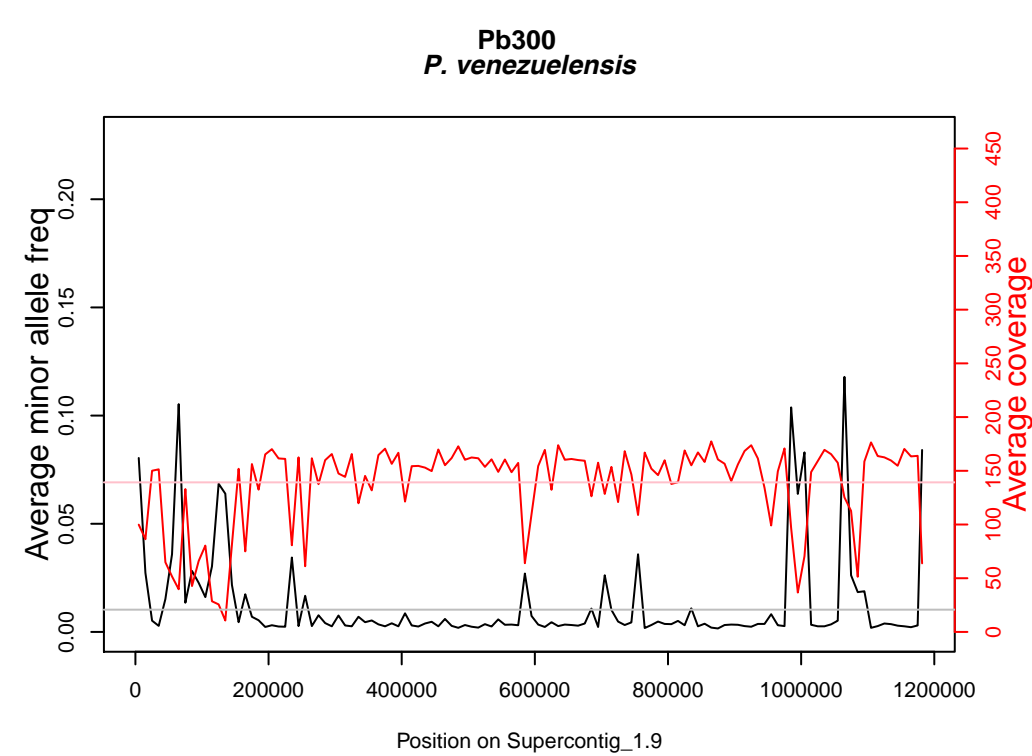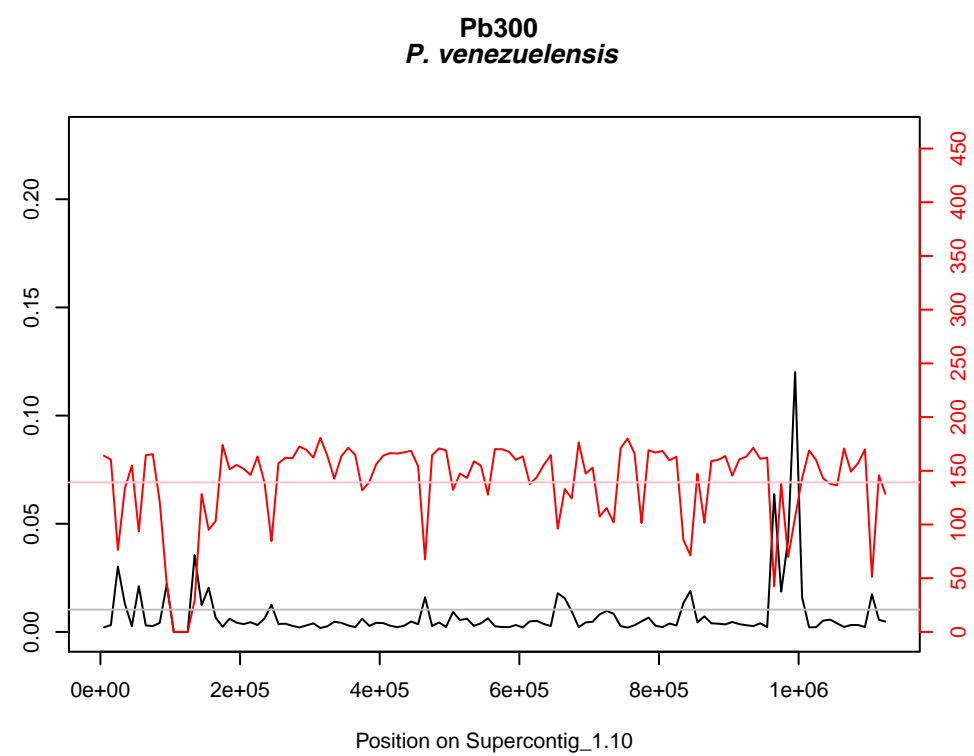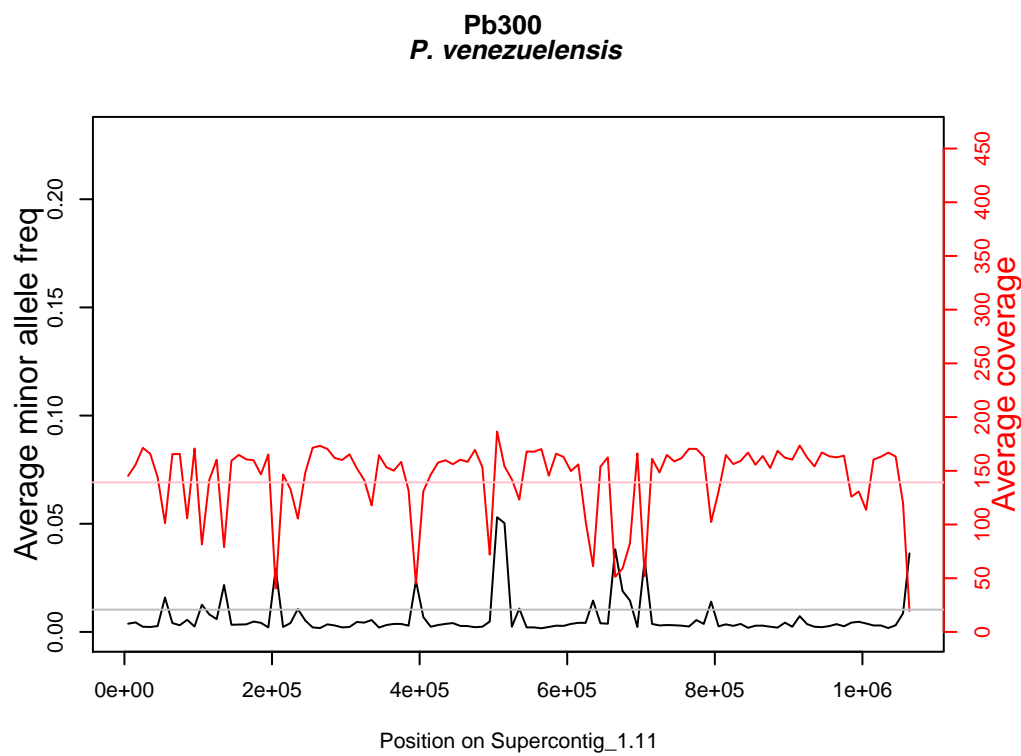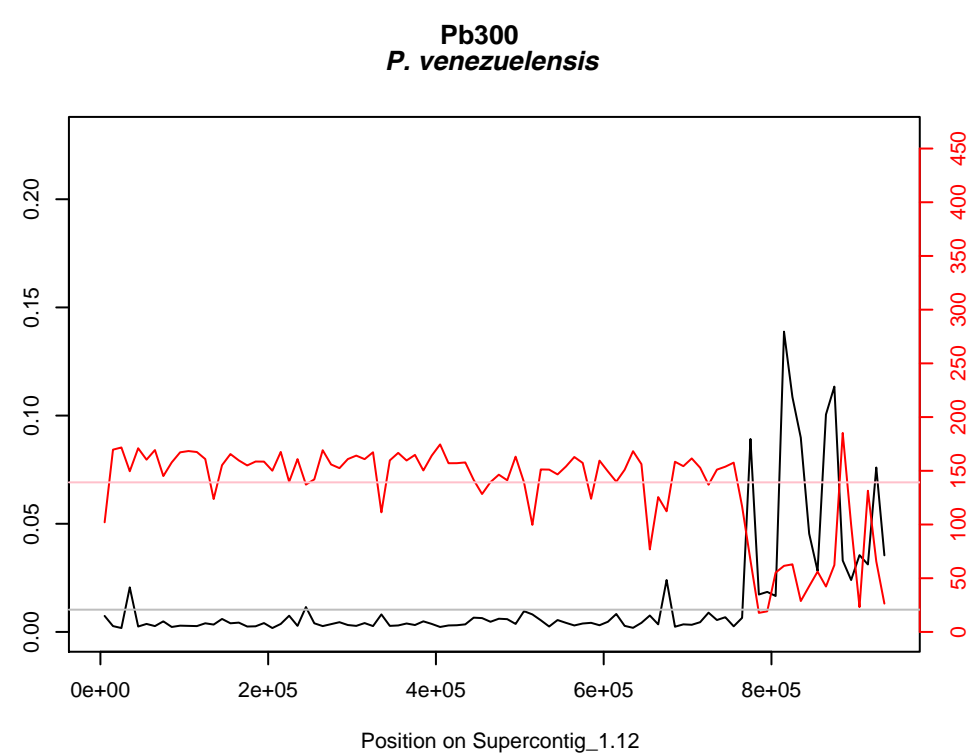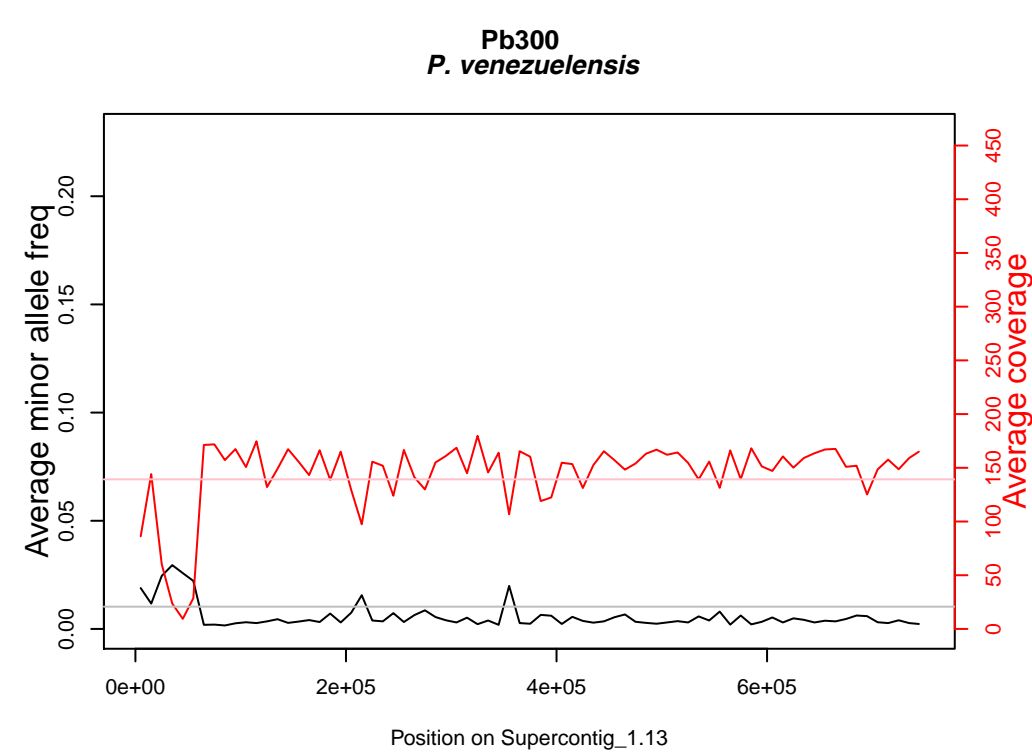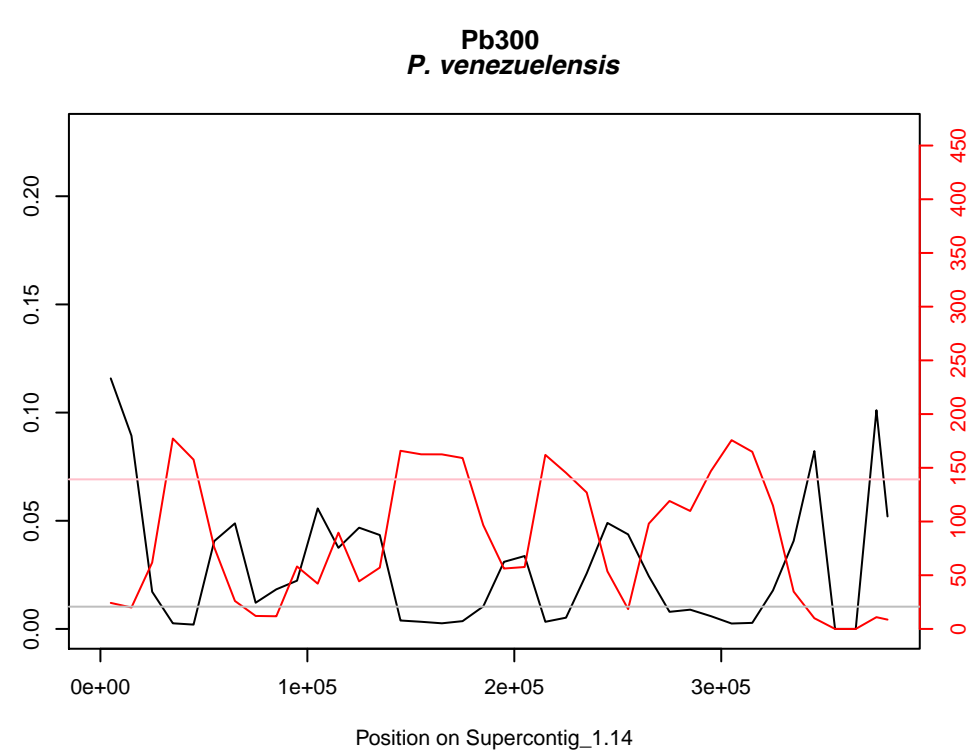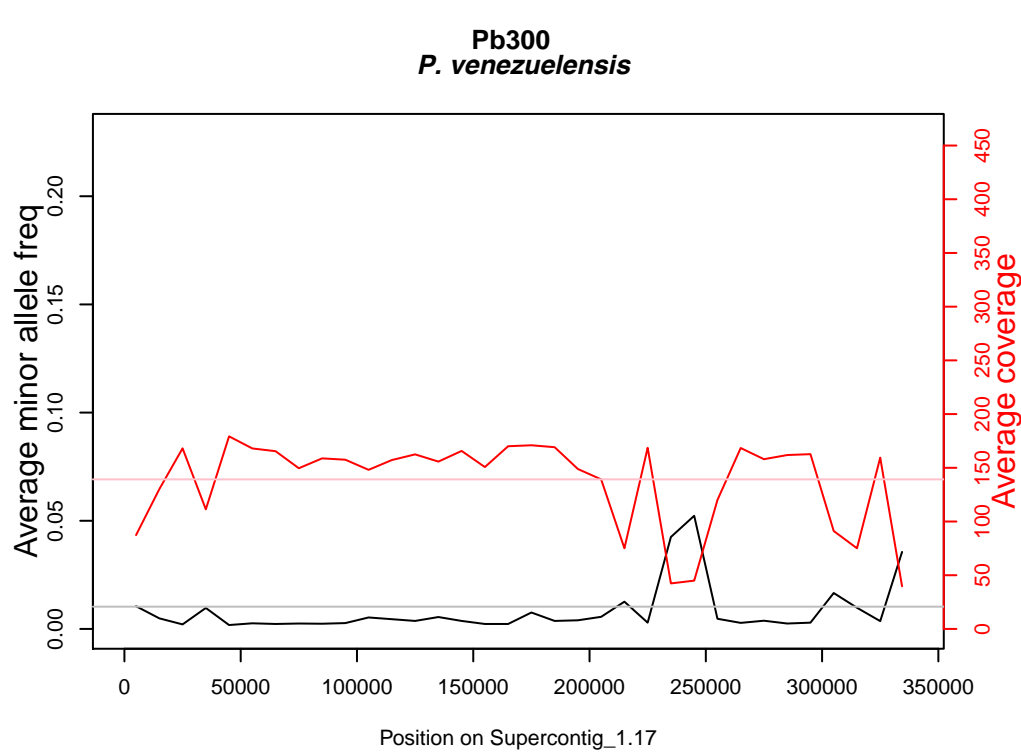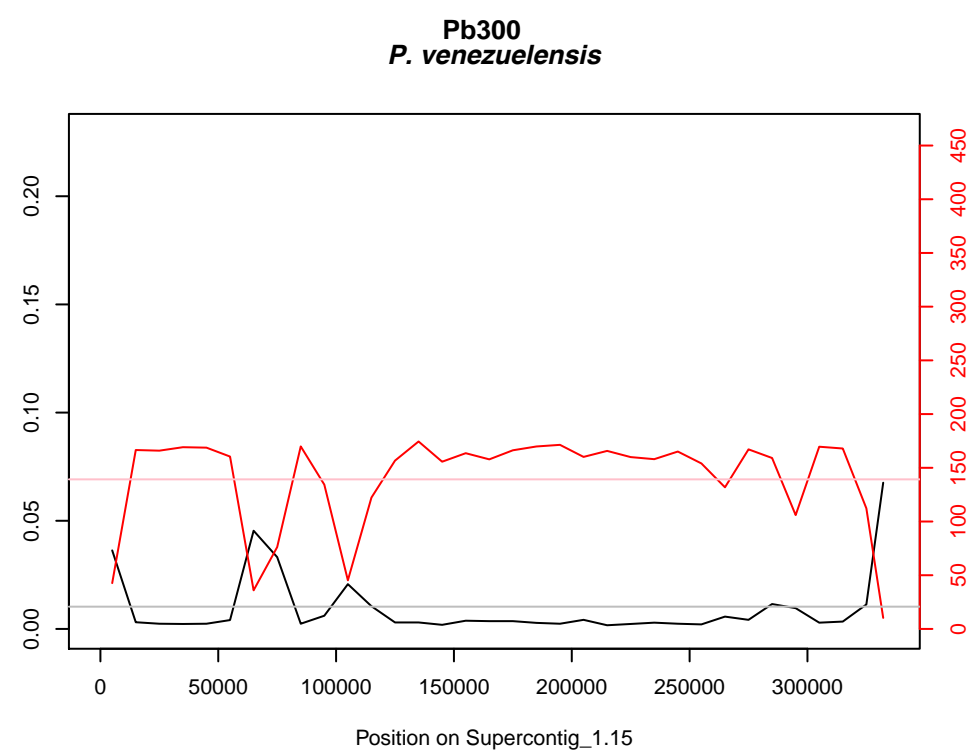

Supplement: FIG S5 [file mbio.01999-20-sf005.pdf]

**Supercontig 1.1**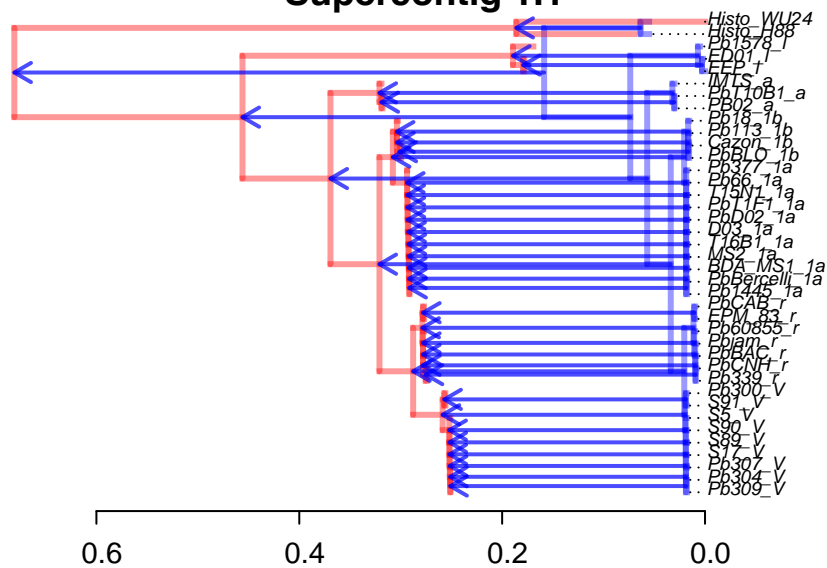**Supercontig 1.2**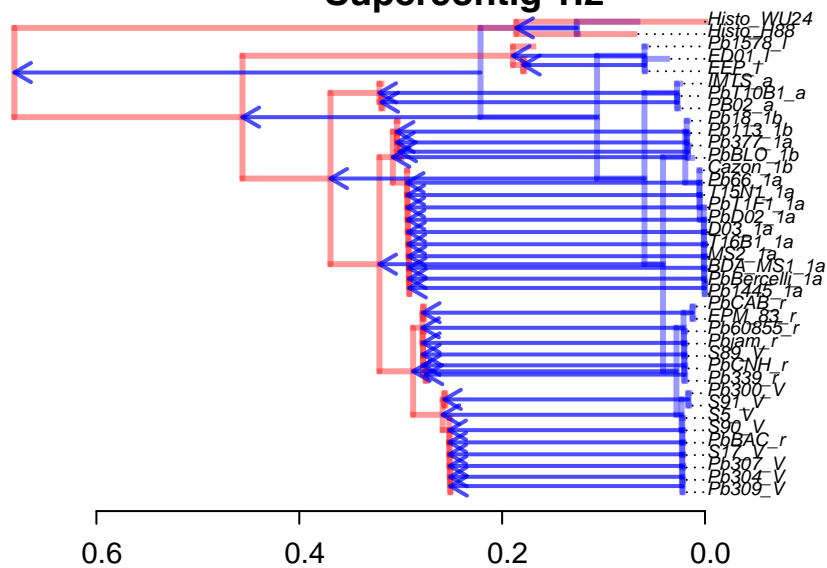**Supercontig 1.3**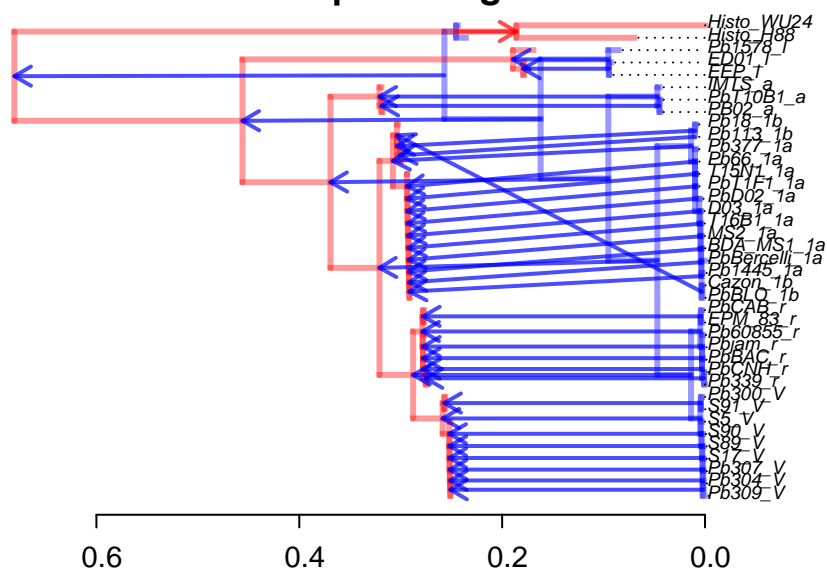**Supercontig 1.4**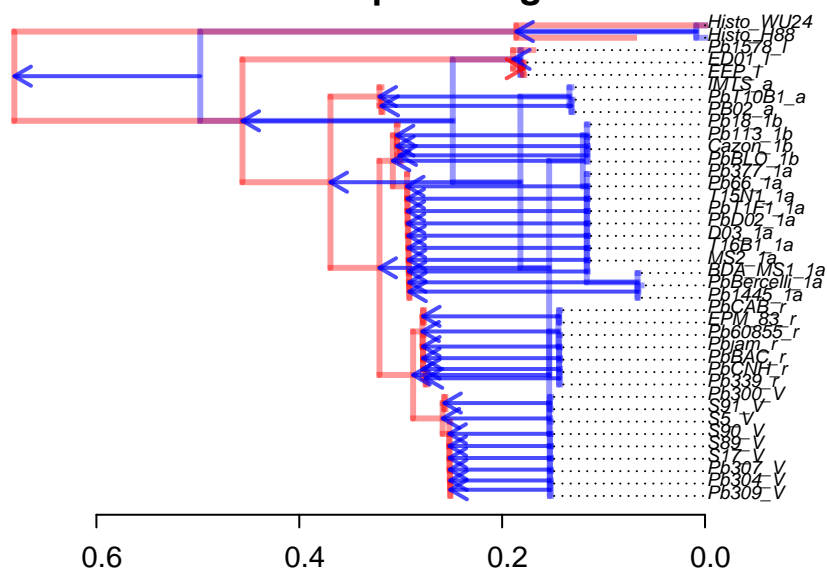**Supercontig 1.5**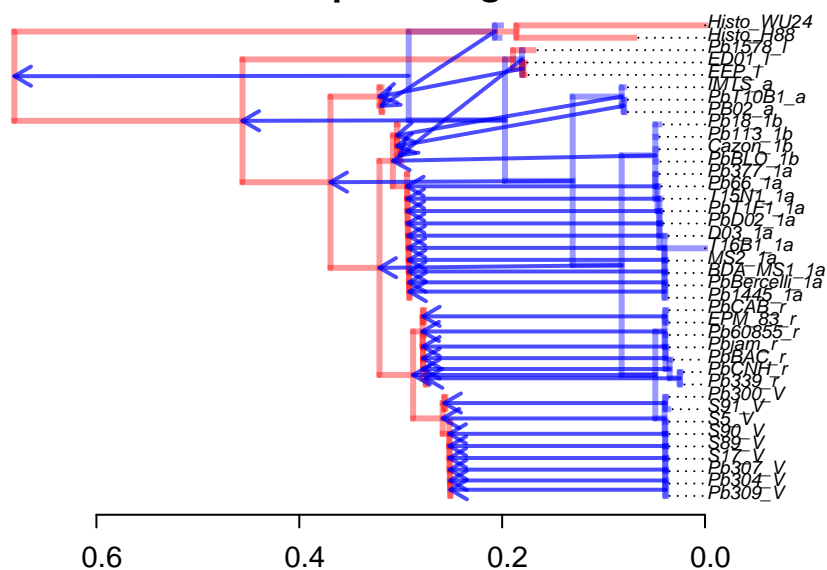**Supercontig 1.6**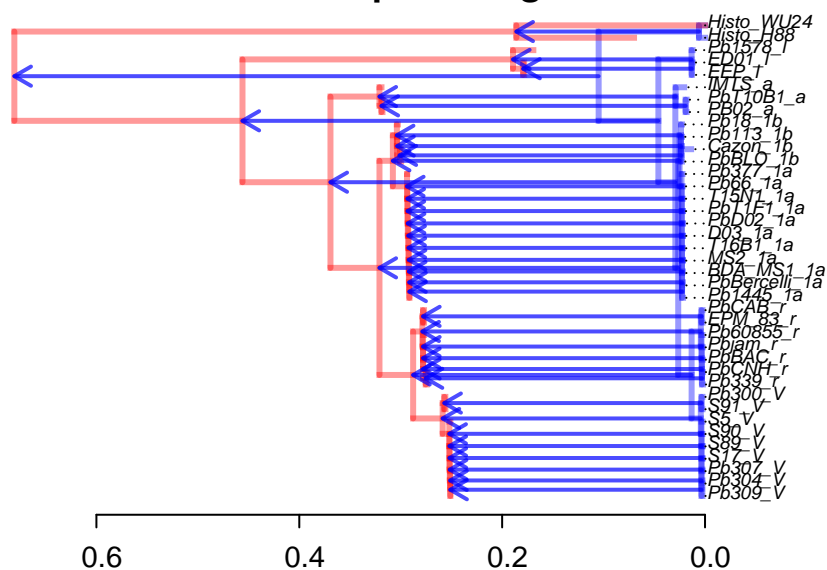

Supplement: FIG S6 [file mbio.01999-20-sf006.pdf]

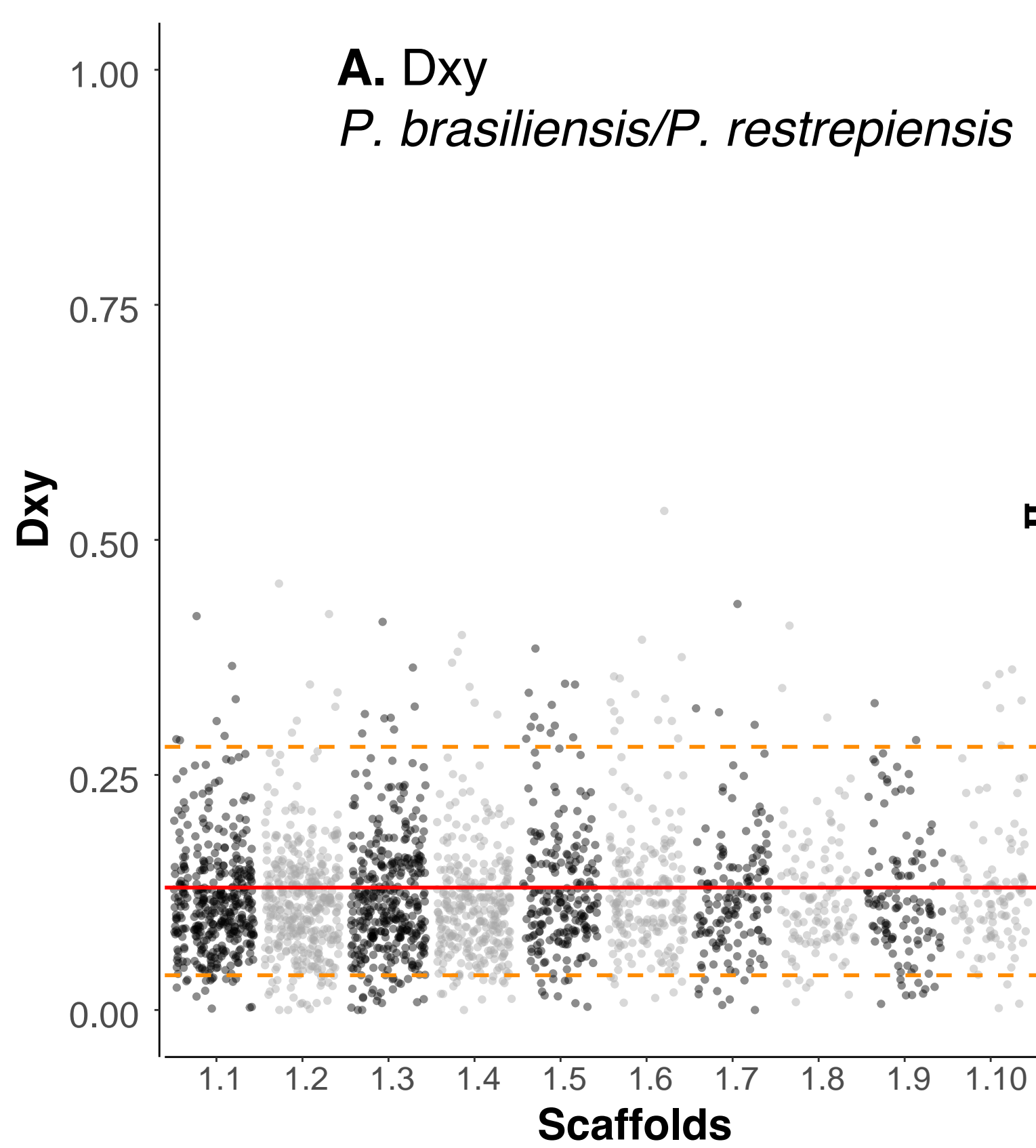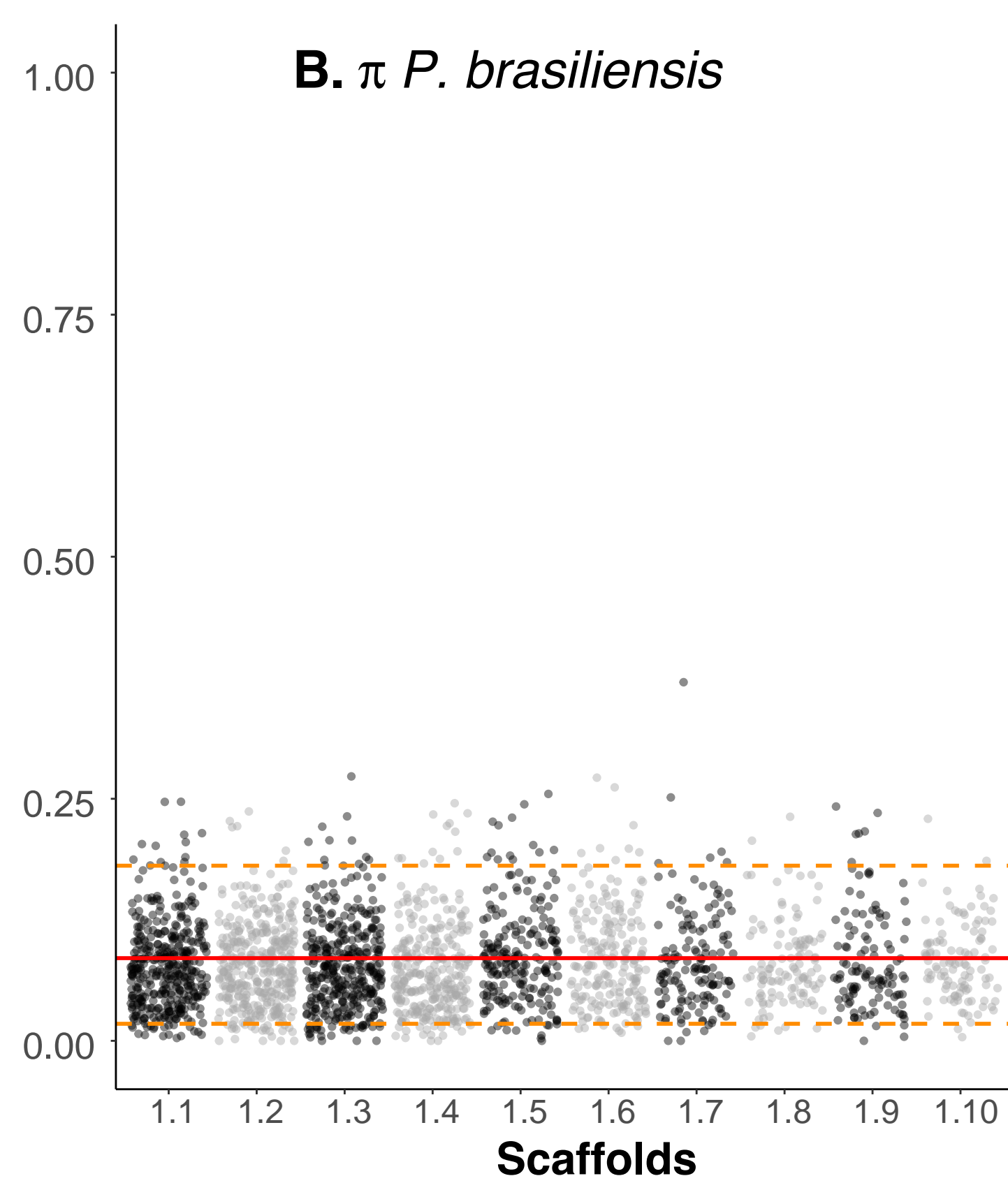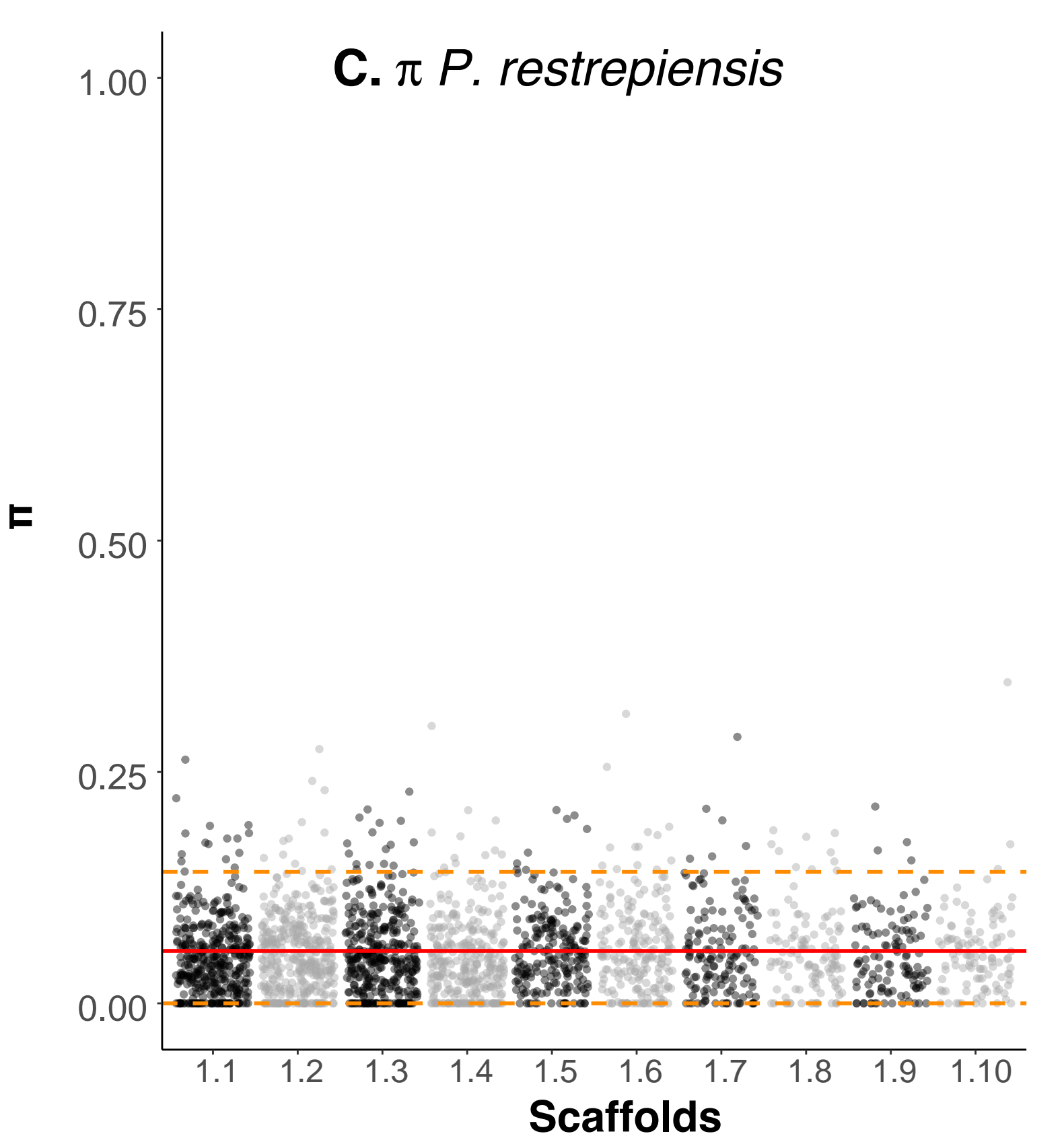

Supplement: FIG S9 [file mbio.01999-20-sf009.pdf]
