# Supplementary material for: Paracoccidioides Genomes Reflect High Levels of Species Divergence and Little Interspecific Gene Flow
Source: mBio. 2020 Dec 22;11(6):e01999-20. doi: 10.1128/mBio.01999-20 (PMC8534288; doi:10.1128/mBio.01999-20)
Supplement: FIG S7 [file mbio.01999-20-sf007.pdf]

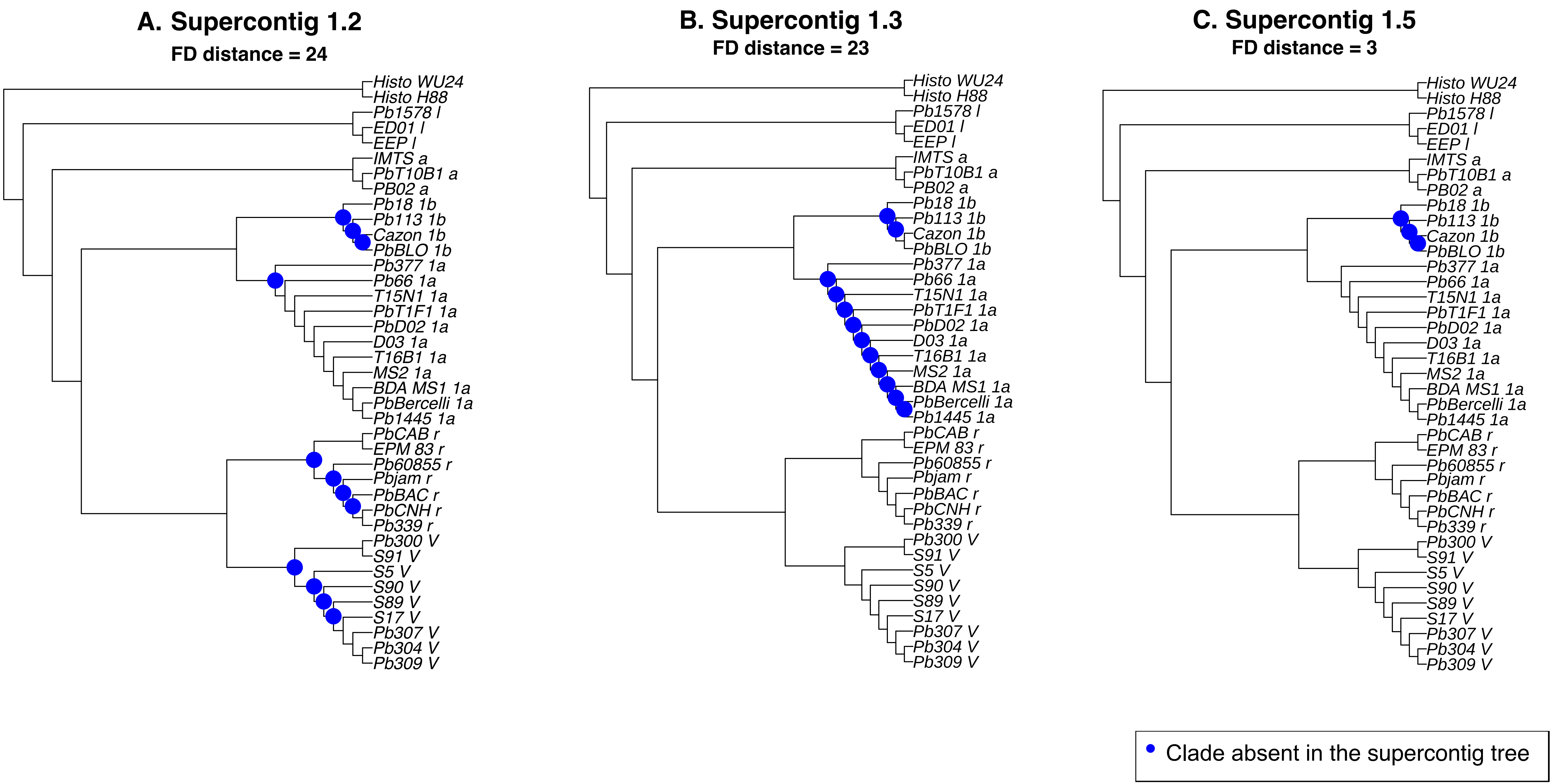
A. Supercontig 1.2
FD distance = 24
B. Supercontig 1.3
FD distance = 23
C. Supercontig 1.5
FD distance = 3
Histo WU24
Histo H88
Pb1578 I
ED01 I
EEP I
IMTS a
PbT10B1 a
PB02 a
Pb18 1b
Pb113 1b
Cazon 1b
PbBLO 1b
Pb377 1a
Pb66 1a
T15N1 1a
PbT1F1 1a
PbD02 1a
D03 1a
T16B1 1a
MS2 1a
BDA MS1 1a
PbBercelli 1a
Pb1445 1a
PbCAB r
EPM 83 r
Pb60855 r
Pbjam r
PbBAC r
PbCNH r
Pb339 r
Pb300 V
S91 V
S5 V
S90 V
S89 V
S17 V
Pb307 V
Pb304 V
Pb309 V
Clade absent in the supercontig tree
