# Supplementary material for: Paracoccidioides Genomes Reflect High Levels of Species Divergence and Little Interspecific Gene Flow
Source: mBio. 2020 Dec 22;11(6):e01999-20. doi: 10.1128/mBio.01999-20 (PMC8534288; doi:10.1128/mBio.01999-20)
Supplement: FIG S8 [file mbio.01999-20-sf008.pdf]

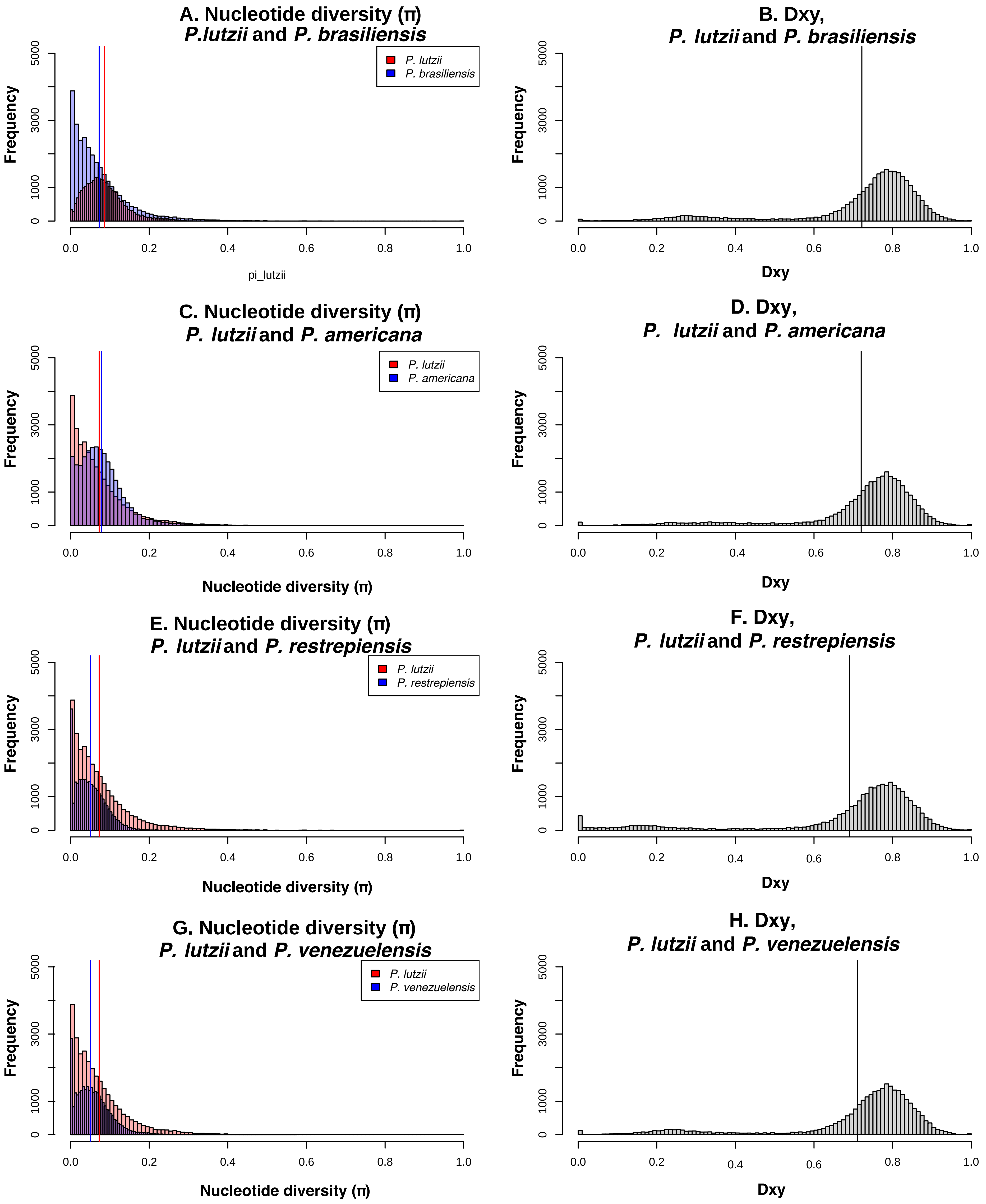
A. Nucleotide diversity (π)
P.lutzii and P. brasiliensis
P. lutzii
P. brasiliensis
Frequency
pi_lutzii
B. Dxy,
P. lutzii and P. brasiliensis
Frequency
Dxy
C. Nucleotide diversity (π)
P. lutzii and P. americana
P. lutzii
P. americana
Frequency
Nucleotide diversity (π)
D. Dxy,
P. lutzii and P. americana
Frequency
Dxy
E. Nucleotide diversity (π)
P. lutzii and P. restrepiensis
P. lutzii
P. restrepiensis
Frequency
Nucleotide diversity (π)
F. Dxy,
P. lutzii and P. restrepiensis
Frequency
Dxy
G. Nucleotide diversity (π)
P. lutzii and P. venezuelensis
P. lutzii
P. venezuelensis
Frequency
Nucleotide diversity (π)
H. Dxy,
P. lutzii and P. venezuelensis
Frequency
Dxy
