# Supplementary material for: Paracoccidioides Genomes Reflect High Levels of Species Divergence and Little Interspecific Gene Flow
Source: mBio. 2020 Dec 22;11(6):e01999-20. doi: 10.1128/mBio.01999-20 (PMC8534288; doi:10.1128/mBio.01999-20)
Supplement: FIG S2 [file mbio.01999-20-sf002.pdf]

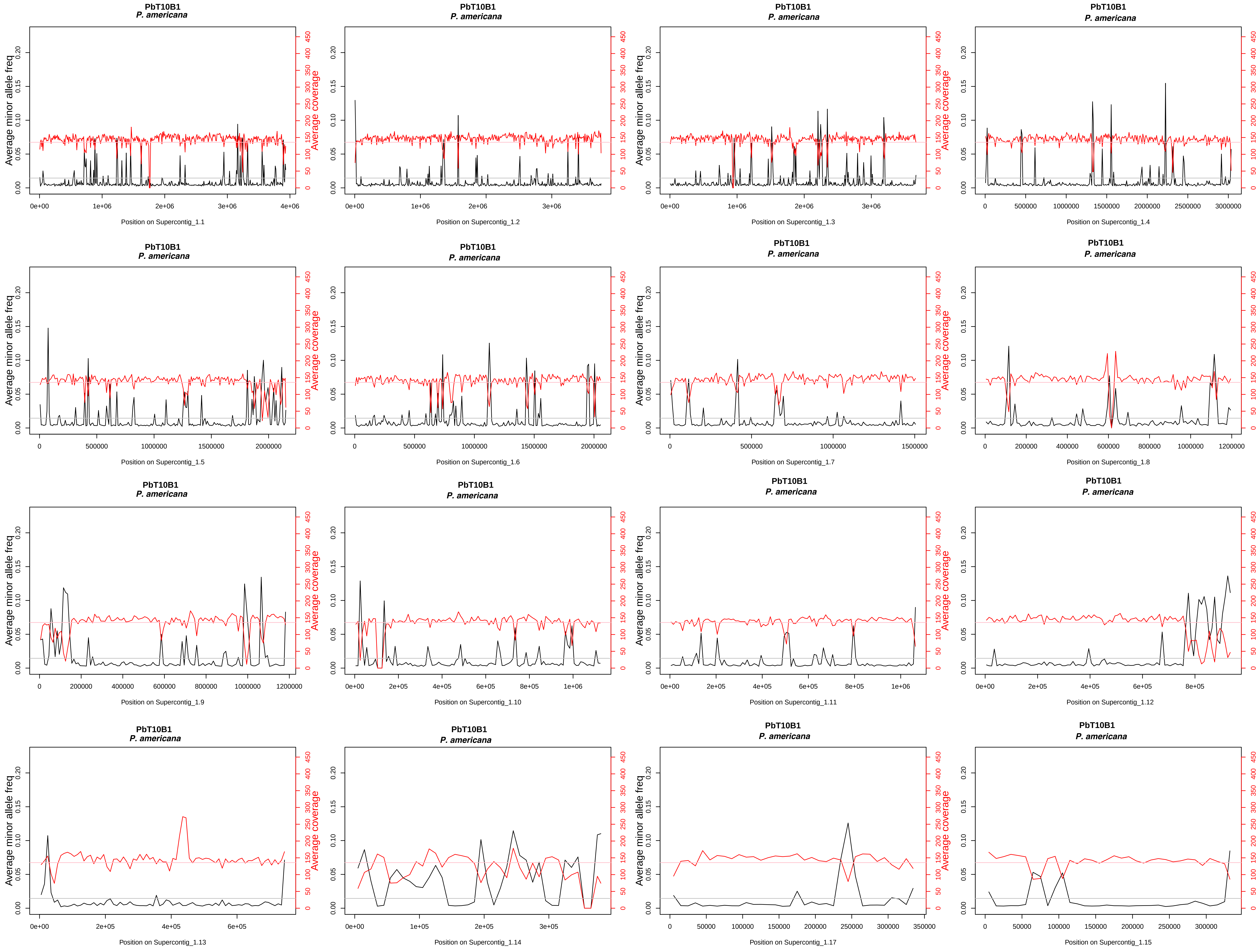
PbT10B1
P. americana
Average minor allele freq
Average coverage
Position on Supercontig_1.1
PbT10B1
P. americana
Position on Supercontig_1.2
PbT10B1
P. americana
Average minor allele freq
Average coverage
Position on Supercontig_1.3
PbT10B1
P. americana
Position on Supercontig_1.4
PbT10B1
P. americana
Average minor allele freq
Average coverage
Position on Supercontig_1.5
PbT10B1
P. americana
Position on Supercontig_1.6
PbT10B1
P. americana
Average minor allele freq
Average coverage
Position on Supercontig_1.7
PbT10B1
P. americana
Position on Supercontig_1.8
PbT10B1
P. americana
Average minor allele freq
Average coverage
Position on Supercontig_1.9
PbT10B1
P. americana
Position on Supercontig_1.10
PbT10B1
P. americana
Average minor allele freq
Average coverage
Position on Supercontig_1.11
PbT10B1
P. americana
Position on Supercontig_1.12
PbT10B1
P. americana
Average minor allele freq
Average coverage
Position on Supercontig_1.13
PbT10B1
P. americana
Position on Supercontig_1.14
PbT10B1
P. americana
Average minor allele freq
Average coverage
Position on Supercontig_1.17
PbT10B1
P. americana
Position on Supercontig_1.15
